# Predicting Epitope Candidates for SARS-CoV-2

**DOI:** 10.3390/v14081837

**Published:** 2022-08-21

**Authors:** Akshay Agarwal, Kristen L. Beck, Sara Capponi, Mark Kunitomi, Gowri Nayar, Edward Seabolt, Gandhar Mahadeshwar, Simone Bianco, Vandana Mukherjee, James H. Kaufman

**Affiliations:** 1 AI and Cognitive Software, IBM Almaden Research Center, San Jose, CA 95120, USA; 2 NSF Center for Cellular Construction, San Francisco, CA 94158, USA

**Keywords:** SARS-CoV-2, epitope, computational biology, mutational analysis, immunology

## Abstract

Epitopes are short amino acid sequences that define the antigen signature to which an antibody or T cell receptor binds. In light of the current pandemic, epitope analysis and prediction are paramount to improving serological testing and developing vaccines. In this paper, known epitope sequences from SARS-CoV, SARS-CoV-2, and other Coronaviridae were leveraged to identify additional antigen regions in 62K SARS-CoV-2 genomes. Additionally, we present epitope distribution across SARS-CoV-2 genomes, locate the most commonly found epitopes, and discuss where epitopes are located on proteins and how epitopes can be grouped into classes. The mutation density of different protein regions is presented using a big data approach. It was observed that there are 112 B cell and 279 T cell conserved epitopes between SARS-CoV-2 and SARS-CoV, with more diverse sequences found in Nucleoprotein and Spike glycoprotein.

## 1. Introduction

Since the first case of COVID-19 was reported in December 2019, the prevalence of SARS-CoV-2 has grown at an incredible pace, resulting in a worldwide pandemic. The scale and rate of spread of the disease continue to grow. As a consequence, humans have had to drastically change their lifestyle, and scientists and clinicians have been presented with great challenges to address such a paramount public health issue. The research needs primarily include expansion of our fundamental knowledge about SARS-CoV-2 in the context of other genomically similar Coronaviridae, analyzing individual and population level immune responses, to model the disease spread and to improve and support vaccine development.

Adaptive immunity comprises humoral and cell-mediated immunity and provides the major mechanisms for individuals to recognize and mitigate foreign agents or antigens. Humoral immunity is driven by B cell lymphocytes and occurs when foreign materials, i.e., antigens from an extratracellular agent such as bacteria or virus, occurs in the body. After introduction to an antigen, B cells differentiate, leading to the creation of memory and effector B cells. Cell-mediated immunity, however, does not depend on antibodies for its adaptive immunity and is instead controlled by mature T cells, macrophages, and cytokines functioning in response to an antigen.

An epitope is a small site on an antigen where a complementary antibody or T cell receptor can specifically bind. The corresponding antigens vary between 8 and 17 amino acids in length, and each epitope is defined by a unique sequence of amino acids. Epitopes are broadly classified into two structural categories: linear epitopes that are continuous amino acid sequences, and non-linear or conformational epitopes that are discontinuous peptides on the unfolded sequence. Non-linear epitopes may only be recognized by the immune system depending on neighboring loci. In this investigation, we focus on linear epitopes. There are two types of epitopes: T cell epitopes and B cell epitopes. B cell epitopes are solvent-exposed portions of peptides on the surface of an antigen that bind to secreted and cell-bound immunoglobulins, i.e., the ones to which antibodies bind [1].

T cell epitopes are further separated into Class I (usually 8–11 amino acids long) and Class II based on their presentation by the major histocompatibility complex (MHC) molecule for recognition by two distinct subsets of T cells: cytotoxic CD8+ and helper CD4+ T cells, respectively [1]. Additionally, their peptide editor mechanism differs: MHC class I uses tapasin, whereas MHC class II uses human leukocyte antigen DM (HLA-DM) [2]. There are also subtle conformational differences in peptide loading and exchange [2]. Differentiating between MHC classes has proven difficult, and often computational methods are limited in their accuracy [3], necessitating a focus on sequence homology when available. Epitope-based vaccines contain isolated B cell or T cell epitopes and typically contain multiple distinct epitopes in order to increase the effectiveness of the vaccine against viral evolution. Epitope-based vaccines must be developed with considerations for antigenicity, solvent accessibility, allergenicity, and toxicity, as shown in Li et al. [4].

With regard to linear epitopes, there are several tools that aim to predict epitope sequence and structure using a multitude of machine learning (ML) methods, which achieve varying levels of accuracy. EpitopeVec [5] predicts linear B cell epitopes using deep protein sequence embeddings and achieves greater than 80% accuracy in 5-fold cross-validation experiments. BepiPred-2.0 [6] leverages a random forest algorithm trained on epitopes annotated from antibody–antigen protein structures and is also leveraged in Griffoni et al. [7]. BepiPred-2.0 [6] has been accepted as state of the art in the community; however, it was observed to decrease to 60% accuracy when evaluated against testing data it had not been trained against [5]. EpiBuilder [8] expands on the results from BepiPred-2.0 to provide epitope assembly, classification, and search in a preoteome-wide processing approach. ABCPred [9] uses a feed-forward (FNN) and recurrent neural network (RNN) to predict B cell epitope regions in an antigen sequence with an overall prediction accuracy of approximately 65%. SVMTrip [10] is named as such because it leverages a support vector machine to combine tri-peptide similarity and propensity scores to detect linear B cell epitopes with an area under the curve (AUC) of 0.702. EpiDope [11] uses a deep neural network to predict B cell epitope regions on individual protein sequences and was trained from 25 K experimentally confirmed epitope and non-epitope regions. In our work, we provide an alternative to these ML-based methods for BCE (B cell epitope) prediction that instead leverages sequence homology and aims to provide increased explainability of epitope detection and evolution, which can be particularly relevant in the case of emerging infectious diseases such as SARS-CoV-2, where black box models may be limited. For T cell prediction, we use sequence homology approaches; however, to predict MHC class, we use netMHC4.0 [12] on our set of epitopes.

In our work, we focus on Nucleoprotein and Spike glycoprotein, as they have been identified as the main proteins of interest in SARS-CoV-2 [4,7,13,14,15,16]. The structural Nucleoprotein (N) is the main virion component. It encapsulates the negative-stranded RNA. The viral genomics RNA and the N protein assemble into the ribo-nucleoprotein, which interacts with the membrane (M) protein and is packaged into virions [13]. The N protein is also involved in other viral functions, including mRNA transcription, replication [17,18], and immune regulation [14,19,20]. For other RNA viruses, including influenza, N sequence is often used for species identification [21]. These N proteins have two functionally distinct conserved structural domains responsible for RNA binding: the N-terminal RNA-binding domain (NTD) and the C-terminal domain (CTD), with the latter also involved in dimerization [22]. These conserved domains are connected by an intrinsically disordered region (IDR) called linker and flanked by intrinsically disordered regions (IDRs). Due to the large disordered regions, the whole N protein structure has not been resolved yet, but the N-NTD and N-CTD domains have been solved at high resolution for different human-infecting Coronaviridae [23,24,25,26,27], including SARS-CoV-2 [28,29,30,31].

The Spike glycoprotein (S) is important, as it plays a vital role in binding, fusion, and entry into the host cell  [15,16,32,33,34,35,36,37]. The S protein is a homotrimeric class I fusion protein, and each protomer comprises two functional subunits, S1 and S2. The S protein is found on the surface of the viral membrane in the metastable prefusion configuration and undergoes major conformational changes to allow membrane fusion with the host cell [32,33,34,36,37,38,39]. This process occurs after the receptor binding domain (RBD) in S1 binds to the host cell receptor. The RBD is flanked by the NTD and the CTD (CTD1, CTD2) [32]. There are two cleavage sites, and one of them separates S1 from S2 fragment. During cell entry, S1 dissociates and S2 refolds into a stable post-fusion state [40,41,42]. S2 comprises the fusion peptide (FP), the FP proximal region (FPPR), the heptad repeat 1 (HR1), the central helix (CH), the connector domain (CD), the heptad repeat 2 (HR2), a transmembrane domain (TM), and the cytoplasmic tail (CT). Comparison between pre- and post-fusion structures suggests that HR1 transitioning movements allow insertion of the FP into the host cell membrane and folding back of HR2 [32]. In the postfusion structure, HR1 and CH form a ≈ 180 Å  extended, three-stranded coiled coil.

There were two primary motivations for studying and predicting epitopes in SARS-CoV-2 by leveraging known epitopes of other Coronaviridae. First, understanding the homology of epitope sequences between different related viruses could provide valuable insight for developing better epidemiological models that could account for adaptive immune response developed from exposure to similar pathogens. Second, the adaptive immune system of two individuals infected by the same organism may learn different peptide sequences and, therefore, produce antibodies for different epitopes. Conversely, as some protein domains are common between organisms, an adaptive immune response developed on the basis of exposure to one organism may sometimes increase risk against a different infectious pathogen [43]. Identification of common epitopes between related viruses may also help pharmaceutical research groups identify therapeutics more quickly by looking at existing treatments for other organisms. Identifying conserved targets may also aid vaccine design and development.

To further the understanding of the adaptive immune response to SARS-CoV-2 and to increase our understanding of its genotypic and phenotypic homology with SARS, we aimed to identify and investigate the presence and evolution of SARS-CoV-2 epitopes, specifically Nucleoprotein (N) and Spike glycoprotein (S). We present our analysis of 11 different SARS-CoV-2 proteins amounting to 28 K unique protein sequences using in silico methods for epitope prediction based on sequence homology. We used data from ancestral lineages as well as eight variants of concern and of interest to show the relevance of results specifically for the Delta variant, thus underscoring the stability of top epitopes. Beyond this, we analyze how the occurrence of different groups of epitopes vary by host. Specific emphasis is placed on detailing Nucleoprotein and Spike glycoprotein findings with structural depictions, as these proteins are of particular medical interest in human health [7]. We believe that even though this approach has been applied to SARS-CoV-2, it can be adopted as a more general guidance to study newly emerging viruses.

## 2. Methods

### 2.1. Description of Data Used

Data for this study were obtained primarily from two sources. Coronaviridae epitope data were retrieved from the Immune Epitope Database and Analysis Resource (IEDB) on 20 July 2020 and again in April 2021 [44]. The IBM Research Functional Genomics Platform (FGP) [45] with semi-supervised SARS-CoV-2 genome annotation method [46] was used to identify and retrieve the protein sequences, domain sequences, and genome accessions from January 2020 to April 2021. This includes ancestral lineage as well as sampled genomes spanning eight variants of concern and of interest, as described by Beck et al. [46]. Using this cohort of genome sequences and, separately retrieved, most frequent S sequences for Delta, we specifically demonstrate the mutational stability of top ten epitopes with variants of concern as well. For access to data used in this study, please reference Data Availability (Section 5).

#### 2.1.1. Immune Epitope Database

From IEDB, linear B cell and T cell epitopes for all Coronaviridae were downloaded as of April 2021. These data were filtered for *positive assays* only, i.e., epitopes known through confirmed laboratory experiments. No filters for host, MHC restrictions, or diseases were applied. For epitope sequences in IEDB that were found originally in other Coronaviridae, the identification of those sequences in SARS-CoV-2 was based on observation of the exact sequence in SARS-CoV-2 proteins in FGP (see Section 2.3 and Section 2.4).

At the time of download in 2020, the data contained 601 linear T cell epitopes with positive assays across 10 proteins and 23 organisms, and 586 linear B cell epitopes with positive assays across 12 proteins and 30 organisms. There were 28 column descriptors, of which the following were extracted for this study:

1. Epitope ID;
2. Parent Protein;
3. Parent Protein Accession;
4. Antigen Name;
5. Epitope Description (Sequence).

From IEDB, we retrieved all distinct (non-redundant) epitope sequences, the name of the protein from which they were found, and the antigen on which it was identified. This was used as ground truth data to locate conserved regions across SARS-CoV-2 proteins and to identify new epitopes not originally in IEDB.

Supplemental Figure S2 quantifies the data availability of IEDB B cell and T cell epitopes by organism and protein. Here, we do not separate the epitopes based on MHC classes they bind to because our methodology does not rely on prediction tools, where this separation can be important to select the correct allele and tools. Additionally, we wanted to see how epitopes cluster based on sequence and position, independent of their MHC Class.

#### 2.1.2. SARS-CoV-2 Genomic Data

The Functional Genomics Platform (https://ibm.biz/functional-genomics) is a comprehensive database and analytics platform that provides (at the time of this work) ∼300,000 pre-annotated bacterial and viral genomes, with over 75 million unique gene sequences, over 57 million protein sequences, and over 263 million functional domains. For SARS-CoV-2, it contains high quality, curated genomic data defined as genomic sequences with <1% Ns (unknown bases), over 29,000 bp, and <0.05% unique amino acid mutations (as described in [46]), along with gene, protein, and functional domain sequences. Moreover, it also provides analytical capabilities such as BLAST over the data. These data, as well as the analytical tools, can be accessed via the Python SDK or the API endpoints provided by FGP after creating a free account.

For this study, we utilized ∼28 K unique SARS-CoV-2 protein sequences and ∼49 K related protein domains and annotations identified from a collection of ∼62 K high quality SARS-CoV-2 genomes from NCBI GenBank [47] and GISAID [48] using the methods described in Beck et al. [46] (full protein and domain sequences can be found with that publication). The proteins and domains identified by FGP are related to the respective source’s genome accession. There are a total of 16 different proteins by name (including hypothetical proteins) for SARS-CoV-2, with 11 proteins annotated per SARS-CoV-2 genome on average. FGP uses md5 hash of a sequence in order to construct its unique identifier (uid). Thus, each protein sequence has an uid used to identify it. At the time of manuscript preparation, an earlier version of FGP’S semi-supervised SARS-CoV-2 algorithm [46] was utilized that had not yet corrected Replicase polyprotein 1a and 1ab sequences; therefore, analysis on those proteins is limited and not shown in detail in this work.

The host metadata used in this work were downloaded from NCBI GenBank [47] and GISAID [48].

### 2.2. Protein Sequence Diversity Analysis

In the reference genomes for SARS-CoV-2, the copy number for each protein is 1 [49,50]. After annotation of 61,850 genomes, evidence for copy number greater than one was found in only 584 genomes (<1%). As some of the analyses described below depends on subsequent multi-sequence alignment to the reference, these genomes were omitted from the analysis.

### 2.3. Identification of Conserved Epitope Sequences

First, known epitope sequences that are present in our protein sequence corpus were identified. In this section, the set of 28K SARS-CoV-2 protein sequences from FGP was utilized to determine conserved epitope sequences from two subsets within IEDB data: a limited set of ground truth SARS-CoV-2 epitopes and epitopes observed in other Coronaviridae. To compare these sequences, the following steps were completed:

- Identification of whether an epitope occurs on a protein sequence and at what locus (amino acid position) using exact string matching functions provided in base Python. For this, the epitope’s parent protein was matched to the SARS-CoV-2 protein, e.g., epitopeA, whose parent protein indicated in IEDB is Nucleoprotein, would be checked for presence on SARS-CoV-2 Nucleoprotein sequences only;
- Calculation of the number of times an epitope occurs on a protein sequence.

The above analysis was performed separately for B cell and T cell epitopes and for all protein names in the set of parent protein names retrieved from IEDB.

### 2.4. Identification of Candidate Epitope Sequences

Next, identification of candidate epitopes was performed, i.e., potential peptides with high sequence homology to the laboratory confirmed epitopes. This would enable accounting for slight changes in known epitope sequences due to evolving protein regions. CANDIDATE epitopes are denoted as NEW in the Supplemental Data.

To this end, we completed a sequence search using BLAST [51] to compare all epitopes downloaded from IEDB (retrieved 07-2020) against all corresponding (with same parent protein name) SARS-CoV-2 protein sequences using FGP’s BLAST service. FGP provides the capability to run BLAST against pre-constructed databases of nucleotide and amino acid sequences. The SARS-CoV-2 amino acid sequence database was selected for this study. Default parameters were used to run BLASTP, and an e-value threshold of 0.01 was chosen, as that indicates a statistically significant match [52].

The steps outlined in Section 2.3 were repeated with the newly identified set of candidate epitopes to find all protein sequences where these epitopes were found, along with the start indices of those epitopes and their frequency per protein sequence. The results were appended to the output from conserved epitope sequence calculation and these were marked as CANDIDATE, and the conserved epitopes were marked as ORIGINAL.

We relied on sequence homology approach and BLAST to identify CANDIDATE epitopes because epitope prediction tools can sometimes be unreliable [3,7].

### 2.5. Identification of T Cell MHC Class I Epitopes

Because T cell MHC Class I epitopes are known to be important in the process of vaccine design [4,53], prediction of MHC Class I for N and S proteins was performed. Here, netMHC4.0 [12] was used with a list of 12 human HLA Class I alleles that have been known to have wide coverage and are listed in Supplemental Data SD2. Peptide lengths between 8 and 14 were allowed, and the rank threshold was set at 0.5 for strong affinity and at 2 for weak affinity (based on netMHC documentation).

The entire set of CANDIDATE and ORIGINAL T cell epitope sequences from S and N proteins was passed as input to netMHC4.0.

The output from netMHC4.0 was parsed to identify peptides with strong and weak affinities to HLA Class I alleles based on the above threshold. Additionally, overlap of these with our top ten T cell epitopes from N and S proteins was calculated as well as their location in structural representations.

Furthermore, for our top ten T cell N and S epitopes, IEDB was manually searched to record how many epitopes have been verified by assays to be MHC Class I.

### 2.6. Summary Statistics

The presence of epitopes on proteins and genomes was studied from the compiled list of ORIGINAL and CANDIDATE epitopes identified in our set of 28 K protein sequences specifically to answer questions such as: What are the most abundant epitopes? Is an epitope found multiple times in a protein sequence? How many epitopes are present on average in a genome?

### 2.7. Epitope Clustering and Classes

To explore how different epitopes relate to one another based on sequence homology and similarity of their loci on the protein sequence, both sequence-based clustering and position-based clustering of epitopes were performed.

For sequence-based clustering, the following steps were performed:

1. Epitopes (both ORIGINAL and CANDIDATE) were grouped based on functional type (T or B) and parent protein;
2. For each group above, the Levenshtein edit distance measure was calculated (using Python) for every epitope–epitope pair. This yields a square matrix with axes labeled by epitope and cell values as the distances between all pairs;
3. Linkage (Python scipy package) was run on the edit distance matrix using ’Euclidean’ metric and ’Single’ method;
4. The resulting linkage matrix was used to compute and plot a dendrogram;
5. A cluster threshold was defined where cluster members had a length normalized edit distance less than one. We color any linkage lineage lines not clustering together in blue.

After clustering epitopes by sequence and extracting the relevant clusters for each protein and epitope type (B or T cell), the occurrence of these clusters on SARS-CoV-2 genomes was investigated. To this end, the dendrogram was combined with a bar plot indicating frequency of occurrence of all epitopes in the cluster combined to which the epitope belongs.

A stacked bar plot was generated to present the distribution of genomes over three categories of original data source (hosts/samples): humans, animals, and environmental. Source data were retrieved from genome metadata files from NCBI and GISAID. These data have also been made available as a table in Supplemental Data SD3.

To obtain position-based clustering of the epitopes, the following steps were completed:

1. B cell and T cell epitopes were divided into separate sets based on parent protein as described above;
2. To remove spurious sequences or assembly errors, only sequences with length within ±10% of the UniProt [54] reference protein sequence for SARS-CoV-2 were considered, e.g., for Nucleoprotein, the length of reference protein is 419 amino acids, and all sequences that were within a ±10% range of that, i.e., between 378 and 461 amino acids in length, were allowed. For SARS-CoV-2 Spike glycoprotein, the reference protein is of length 1273 amino acids and the allowed range was 1146 to 1400 amino acid characters;
3. Furthermore, multi-sequence alignments (MSA) were run for all sequences per protein name relative to one another using MAFFT (v7.431) with the reorder option [55];
4. For each protein, a matrix was then constructed, with the x-axis indicating the amino acid position on the protein and the y-axis indicating the epitopes where the length of the x-axis was the maximum length of the protein sequence;
5. Each cell was then assigned a binary value, i.e., 0 or 1. The cell is assigned 1 if the epitope on that row is found on the position on the protein corresponding with the column index of the cell, e.g., if ‘epitopeA’ is on row 0 and is found on a Nucleoprotein sequence between indices 7 and 15, then index 7 would be filled with 1;
6. After sequences have been aligned in the MSA, the positional information was padded so that all pairwise comparisons are represented with identical start and stop coordinates, and gaps are filled in with dashes. To accommodate this relative positional information generated by an MSA, regular expressions were used to identify the start index of epitope sequences with the allowance for epitopes that span “gapped regions” due to the filling in of sequences in the MSA;
7. Epitope sequences spanning regions of insertion described in step 6 were investigated further for key non-synonymous mutations and their prevalence in this corpus of ORIGINAL and CANDIDATE epitopes;
8. After alignment, the epitopes were identified on the same position within the protein sequences. The bars representing epitopes were colored by their presence across the genomes;
9. After constructing the matrix in steps 2 and 3, ‘single’ linkage was run using method ‘Euclidean’ metric;
10. Finally, the clustermap and dendrogram were plotted using Python’s seaborn library.

### 2.8. Identification of Epitopes on Protein

#### 2.8.1. Mutations in Proteins Affecting Epitopes and Mutation Density

Certain epitopes were not found as exact subsequences on MSA proteins due to filling in of gap regions. In such cases, a regular expression (regex) match was used to find the start position for the epitope. Additionally, these regions identified locations where protein mutations were occurring and were thus studied in greater detail by visualizing MSA results.

Mutations at any given position on the Spike glycoprotein transcript were logged using the MSA results as percentages. The proportion of amino acids matching the reference were measured at every position on the sequence to estimate ‘mutation density’. These were plotted to examine regions of elevated mutagenesis on the protein sequence with respect to the MSA-derived consensus sequence. Mutation density plots were constructed for both Spike glycoprotein and Nucleoprotein transcripts.

#### 2.8.2. Epitope Distribution across Protein

The clustermap generated in Methods Section 2.7 for position-based clustering also serves to highlight where epitopes lie on the protein. However, to gain an amino acid-position level understanding of ‘immunodominance’, the frequency of epitope occurrence at a given position on the protein transcript was visualized. Our epitope samples were split into ORIGINAL and CANDIDATE epitopes. The number of times that an epitope occurred across all genomes was logged. These frequencies were normalized and plotted along with the normalized global median frequency in order to assess regions of immunodominance relative to other regions on the protein transcript. The proportion of immunodominance prescribed to each position by new epitopes was drawn as a stacked area plot atop the proportion of immunodominance prescribed by original epitopes to compare the contributions of each epitope origin to the overall immunodominance level for the position. This graph was constructed for T cell and B cell epitopes on N and S proteins.

To identify epitope relationships within protein functional domains, the domains for each protein were also checked against epitopes for complete sequence homology, i.e., if an epitope sequence exactly matched or was found as a sub-sequence within a domain sequence for the corresponding protein.

#### 2.8.3. Epitope Localization on Protein Structure

In order to visualize the position of the epitopes on the quaternary structure of the Nucleoprotein (N) and the Spike glycoprotein (S) of SARS-CoV-2, the VMD software [56] was used, and the following procedure was performed for both proteins. First, the reference sequence from UniProt (Supplemental Data SD8) was compared with the sequence of the protein structures deposited in the Protein Data Bank [57,58] and then visualized with the epitope positions in the 3D shape of the protein by selecting the protein deposited structure showing 100% identity with the consensus sequence. Specifically, for the N protein, the 1.5 Å resolution X-ray structure of the C-terminal domain (CTD) resolved by Y. Peng et al. [13] was used (PDB ID: 7CE0); for the S protein in the prefusion and postfusion conformations, we used the cryo-EM structure resolved by Y. Cai et al. [32] at 2.9 and 3.0 Å (PDB ID: 6XR8 and 6XRA, respectively).

Furthermore, MHC Class I epitopes of N and S T cell epitopes were visualized separately on the 3D protein structure.

### 2.9. Result Verification

To understand the correctness of our results, a later set of lab-confirmed epitopes (IEDB retrieved 04-2021) was used. We did not rely on comparing results with other epitope prediction tools since this has been benchmarked comprehensively elsewhere [7], and the accuracy with regards to SARS-CoV-2 was determined to be low [3].

The predicted set of epitopes were analyzed against the latest set of epitopes from IEDB. BLAST [51] was used to find the sequence alignment between the predicted and the known epitope sequences found on Spike glycoprotein and Nucleoprotein. For each protein, the epitopes found in B cell and T cell were separated and then each was used to create a BLAST database using the epitope sequences from IEDB. This generated four BLAST databases, one for each protein and cell type. A protein BLAST query was perfomed with the predicted epitopes as query sequences. All default BLAST inputs were used, except the number of alignments was set to one in order to find the IEDB sequence that best matches each predicted sequence. For each protein and cell type, the average percent was identified, and e-value was calculated between the predicted epitopes and known epitopes.

Additionally, even though a complete analysis with the latest variants of concern was out of the scope for this paper, in light of the outbreak and seriousness of the Delta variant of SARS-CoV-2, the two most frequently seen sequences of Spike glycoprotein for Delta variant were retrieved and assessed to identify if our top Spike T cell and Spike B cell epitopes are present on this Spike sequence. The sequences were obtained from [46] and are included in Supplemental files SD1. As seen here [46], these sequences have been found in other variants of concern as well.

## 3. Results

### 3.1. Protein Sequence Diversity Analysis

In this work, a corpus of 61,850 SARS-CoV-2 genomes from NCBI GenBank and GISAID was analyzed. Protein annotations for each were downloaded from FGP. After performing the data sanitation steps outlined in Methods Section 2.2, 584 (<1%) genomes were identified as low quality where, after annotation, protein sequence diversity was >1 for one or more proteins. These were excluded, resulting in a total genome dataset of 61,266. A total of 4595 and 1737 unique protein sequences were observed in S and N sequences, respectively. From this corpus, the number of observed epitopes are indicated in Table 1.

### 3.2. Epitope Sequences

Section 2.3 outlines the procedure followed for identification of conserved epitopes between SARS-CoV-2 protein sequences and other Coronaviridae. As shown in Table 1, 112 B cell and 279 T cell conserved epitopes were identified across all proteins. Of the 112 linear B cell epitopes from Coronaviridae found in SARS-CoV-2 protein sequences, three are originally from SARS-CoV-2 and 109 from SARS. Of the 279 linear T cell epitopes from Coronaviridae that were found in FGP SARS-CoV-2 protein sequences, 221 are originally from SARS-CoV-2 and 58 from SARS.

Additionally, 492 candidate linear B cell (304) and T cell (188) epitopes were identified in SARS-CoV-2 proteins with high sequence similarity to epitopes from IEDB (Table 1). Our results suggest that linear SARS-CoV-2 epitope sequence length varies from 7 to 42 amino acid characters for both ORIGINAL and CANDIDATE epitope sequences.

The top ten (by occurrence) B and T cell epitopes for Spike glycoprotein and Nucleoprotein are listed in Table 2 and Table 3. The criteria for being a top epitope is based only on the number of genomes an epitope is found in. In addition to the epitope sequences, the tables list the start index of epitopes on aligned protein sequences, if T cell epitope belongs to MHC Class I, parent epitope (if the epitope is derived, i.e., CANDIDATE). and other epitopes that cluster with it based on sequence similarity.

In order to test the predicted epitopes and to measure the sequence diversity, the predicted epitope sequences were compared to the known epitopes for each cell type, and the percent match was calculated. We find the average percent identity for Nucleoprotein B cell is 95.05%, Nucleoprotein T cell is 97.75%, Spike B cell is 94.64%, and Spike T cell is 97.40%. Furthermore, range of percent identities for all predicted epitopes within each cell type was analyzed. Figure S1 shows the average and range of percent identity for each epitope class. This shows that, although the averages for all four classes were greater than 90%, the sequence diversity of B cell epitopes was larger than for T cell epitopes.

Additionally, to verify the validity of the top Spike epitopes in the Delta variant, the presence of the top ten B and T cell Spike epitopes was checked on 2 SARS-CoV-2 Delta spike sequences. We found that all epitopes are indeed present on the Delta Spike as well.

Supplemental Figures S16–S21 show the presence of epitopes in genomes and proteins. We observe a distribution where the epitopes are present in large numbers in most entities and then fall off sharply. This may require further investigation into genomes with a low number of identified epitopes, as these genomes may have sequencing errors or other assembly defects, etc. Note that each epitope was found only once on a protein sequence, and each protein had a copy number of one on a genome, thus each epitope was found at most once per genome (if found at all).

**Figure 1:**
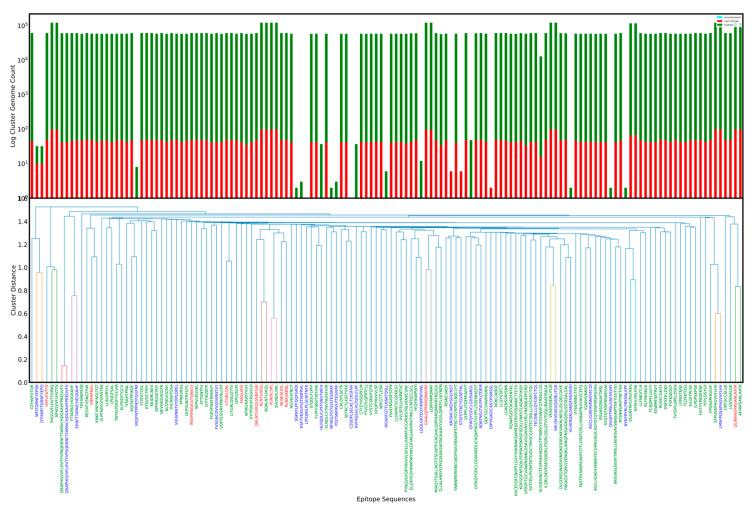
Clustering of Spike glycoprotein T cell Epitopes ORIGINAL+CANDIDATE and their occurrence. The bottom chart is a dendrogram obtained by performing sequence based clustering on T cell S epitopes. The labels along the x-axis in the dendrogram are the epitope sequences and have been assigned colors based on their originating organism: blue if CANDIDATE epitope, red if originally found in SARS, and green if known to be found in SARS-CoV-2. Along the y-axis of the dendrogram is the edit distance score. The edit distance of two sequences lets us know how similar the sequences are to one another. We put a threshold of 1.0 on this edit distance to discover clusters within the epitopes, i.e., epitopes with normalized distance < 1.0 are part of same cluster. In the top part of each figure, the bars align with the epitope labels from the dendrogram. Each bar represents the number of times all members of the cluster to which the epitope belongs are seen across SARS-CoV-2 genomes in our dataset. It is also important to note that the figure is actually a log–log plot of the counts. Furthermore, each bar is stacked based to show genomes sequenced in humans, animals, or environment. We would also like to highlight that low presence in genomes sequenced from environment is not a consequence of epitopes not being found in those genomes, but rather a product of extremely low numbers of high quality genomes from the environment in our dataset. Data used to generate this figure are presented in Supplemental Data SD3.

Figure 1 and Figures S3–S5 show the relations between epitopes from sequence based clustering. Supplemental Data SD3 presents the csv file from which sequence based clustering figures were generated.

In Figure 1, most epitopes are already laboratory confirmed epitopes in SARS-CoV-2. Only one cluster is seen with significant frequency that has more than two epitopes. In Supplemental Figure S5 for Spike B cell, most epitopes are CANDIDATE epitopes, indicating high sequence diversity and potentially many undiscovered or unconfirmed epitopes at the time of analysis. There is only one cluster with significant presence, and even within that, there is only one laboratory confirmed epitope. In Supplemental Figure S4 for Nucleoprotein B cell epitopes, there are no laboratory confirmed epitopes already known to be present in SARS-CoV-2, and top clusters have non-zero CANDIDATE epitopes. This may indicate rapidly evolving regions on proteins leading to evolution of new epitope sequences. In Supplemental Figure S3 for Nucleoprotein T cell epitopes, the cluster with the most significant presence has only SARS and CANDIDATE epitopes.

In all figures noted previously, a significant number of CANDIDATE epitopes are observed. When we compare the presence of clusters against each other, it is observed that there are certain clusters of epitopes that have a much higher weight than other clusters.

### 3.3. T Cell MHC Class I Peptides

T cell MHC Class I epitopes bind to HLA Class I and are known to be important in the process of vaccine design [4,53]; thus, the prediction of MHC Class I for N and S proteins was performed.

As described in Section 2.5, the T cell epitopes of N and S were annotated using netMHC4.0 to identify which epitopes are potentially MHC Class I in their sequence entirety or have peptides regions that bind to HLA Class I alleles. The alleles used are detailed in Supplemental Data SD2. The output from netMHC was filtered to include peptides with strong or weak binding only (defined as rank <= 0.5 and rank > 0.5 and <2, respectively, based on netMHC documentation), and others were discarded. These results are presented in Supplemental Data SD5. NetMHC was run allowing for peptide lengths from 8 to14 mers, as the majority of our peptides were observed to have length < 11, although previous studies indicated those longer than 11 amino acids should be cautiously selected [12].

The alleles from SD5 have been grouped and ordered by their frequency of occurrence and listed in Supplemental Data SD6. For S protein, strong and weak binding was most commonly observed with HLA-A2402. For N protein, strong and weak binding was most commonly observed with HLA-B0702. Interestingly, HLA-A0201 was not found to be the most common allele, as reported in other work [7].

For epitope comparisons with netMHC, we consider matching epitopes as those only with 100% sequence identity. Out of 143 T cell S epitopes, thirteen matched strong binding S peptides and 37 with weak binding S peptides (SD5). Out of 119 T cell N epitopes, only three exhibited strong binding with N peptides and seven with weak binding N peptides (SD5). All of the above matches were found only in ORIGINAL S epitopes and not with any of the CANDIDATE epitopes for both weak and strong. For N epitopes, there was one CANDIDATE epitope, with all others being ORIGINAL for both weak and strong.

From manual inspection of IEDB at the time of writing, all of the top ten T cell epitopes are known epitopes for SARS-CoV-2. In addition, eight of the top ten T cell S epitopes and seven of the top ten T cell N epitopes were identified as MHC Class I through biological assays referenced on IEDB. These have been indicated in Table 2 and Table 3 with an asterisk (*). In contrast, from netMHC in top ten S epitopes, two were strong binding peptides and four were weak binding peptides. For top ten N epitopes, none matched strong binding peptides, and one matched weak binding peptides (Supplemental Data SD7). The above findings are in agreement with [3].

### 3.4. Epitopes on Protein

#### 3.4.1. Mutations in Proteins Affecting Epitopes

Understanding the rate of mutation across epitope sequences can provide insights into waning host immunity and the average period of host reinfection. These insights can also shape our understanding of vaccine efficacy over time. To evaluate this, we computed a multiple sequence alignment (MSA) of unique Spike glycoprotein and Nucleoprotein sequences and evaluated the mutational density across the B cell and T cell epitopes.

**Figure 2:**
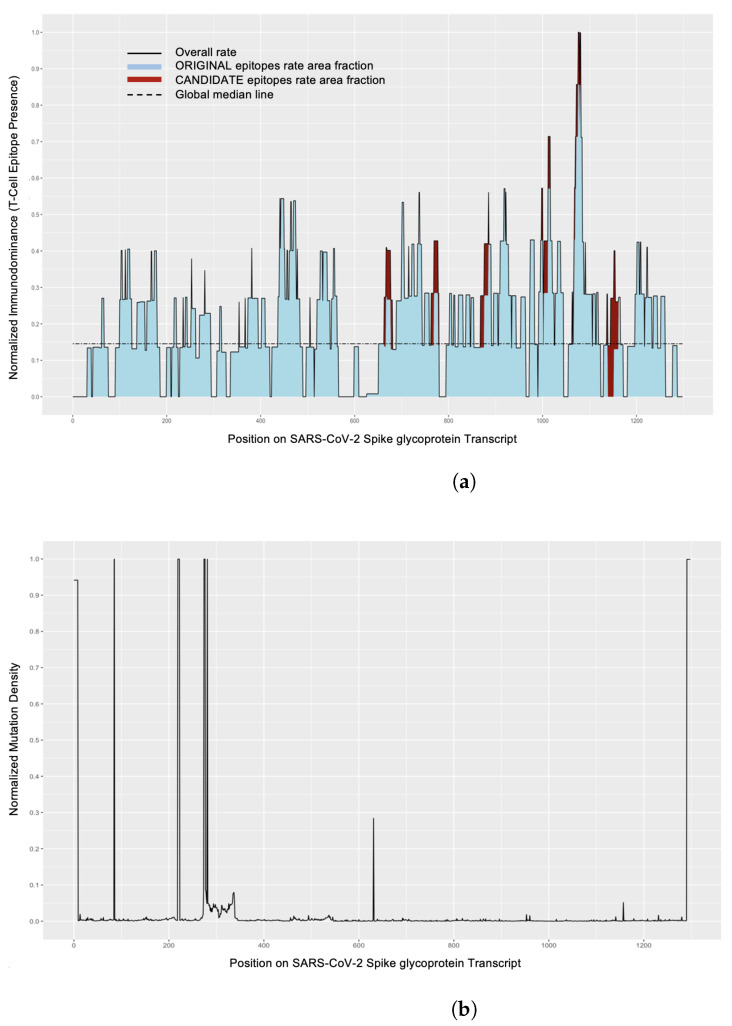
Immunodominance plot and mutagenesis plots. (**a**) Stacked area plot depicting normalized T cell epitope presence across the length of the Spike glycoprotein transcript (total length: 1299 amino acids). The graph is colored by epitope origin, with original epitope rates in blue and newly predicted epitope rates in red. (**b**) Mutation density plot for the Spike glycoprotein; logs normalized mismatch frequency rates across the protein as compared to the consensus sequence.

In Spike glycoprotein (Figure 2),it is observed that specific residue positions have high mutation density when evaluating the consensus sequence obtained after running MSA. In addition, a small region between residue positions 250–350 has slightly higher mutation density on average. In Nucleoprotein, there is slightly above average mutation density around residue position 200.

For Nucleoprotein, there were no epitopes that overlapped with mutations or indels (insertions or deletions). However, for the Spike glycoprotein consensus sequence logo, the most prevalent amino acid by position was observed to be present with a median value of 99.84% (range 0.023%–100%), suggesting high sequence conservation across the protein. However, there was a small amount of mutations or indels in epitope sequences. For example, for epitope WTAGAAAYYVGY at amino acid position 272–288, we observed several key differences from the wild type epitope sequence. We observed a three amino acid insertion in one of our Spike glycoprotein sequences. Additionally, at amino acid position 278, we observed higher variability where the dominant amino acid glycine (G) is present in only 91.41% of sequences, with substitutions to serine (S) or aspartic acid (D) being the most common. This shifts from an aliphatic amino acid to a polar, hydroxylated amino acid or negatively charged acid, respectively, which can then change binding affinity to host proteins.

#### 3.4.2. Epitope Distribution across Protein

**Figure 3:**
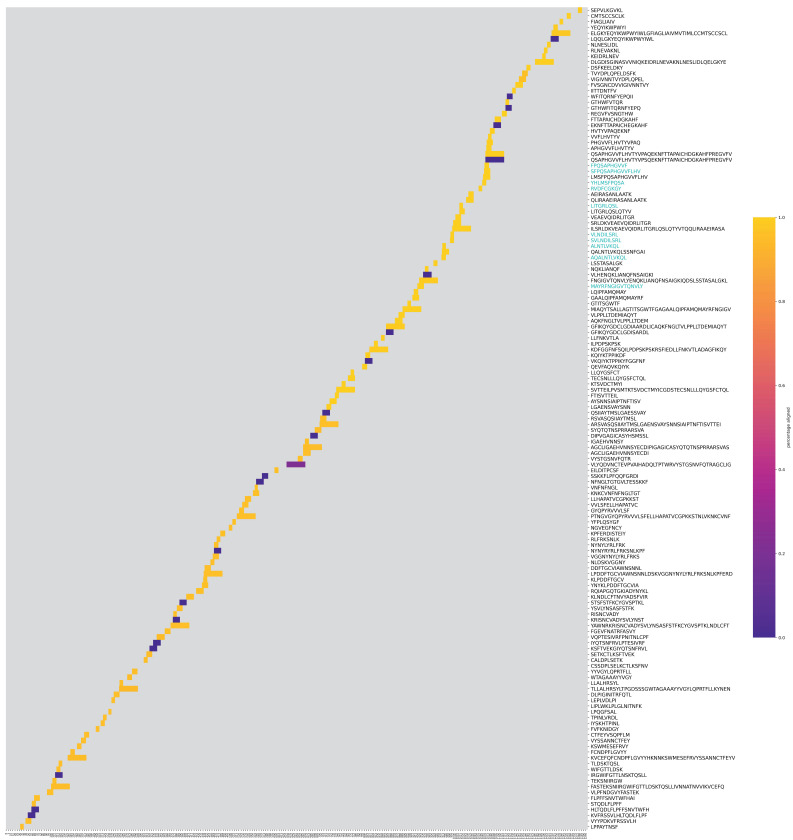
Clustermap obtained after clustering the T cell epitopes of Spike glycoprotein based on the position at which they occur within the protein. The x-axis is the entire length of the protein, which is 1299 in the case of S. Along the y-axis, every row represents one epitope. The color scheme is defined by using a color map that assigns colors to each row depending on occurrences of the epitope across all genomes. The y-axis labels on the right-hand side are colored cyan to indicate an epitope from the top ten list. Data used to generate this figure are present in Supplemental Data SD4.

The immunodominance Figure 2a, Figures S9, S12, and S13 show the presence of T cell and B cell epitopes on Spike glycoprotein and Nucleoprotein proteins. Mutation density plots for S and N proteins are in Figure 2, Figures S10 and S14. We also plot the presence of unknown amino acid by position in Figures S11 and S15. As discussed in Section 2.8.1, we plot the epitopes on each protein and cluster them by position. Figure 3 shows the position of T cell epitopes on S. These plots relay more granular information regarding each epitope and its presence in our genome set and on the protein, along with its positional neighbors, as compared to immunodominance plots, which show aggregate data for all epitopes. Where the slope is high (an increase in differing epitopes at a similar position), the epitope sequences are more diverse and changing most rapidly. Data used to generate Figure 3 and Figures S6–S8 are present in Supplemental Data SD4.

In Figure 2a, an almost even distribution of T cell epitopes on Spike glycoprotein is observed; however, there is an uptick at the 1063 aa position. In addition, there are fewer CANDIDATE epitopes, as already noted in Section 3.1. By simultaneously looking at Figure 3, it is observed that the most prevalent T cell epitopes on Spike are in the S2 region, and three contribute to the uptick along the 1063 aa region.

Supplemental Figure S9 reveals a large number of CANDIDATE B cell epitope sequences on Spike glycoprotein and significant regions on S1 with no epitopes present (also supported in Supplemental Figure S8). From the latter, we can again see that most prevalent Spike B cell epitopes are found on the S2 region.

For Spike, as shown in Figure 2a and Figure S9, the presence of both T cell and B cell epitopes along S2 correlates with lower mutation density in S2.

Supplemental Figure S12 shows immunodominance for Nucleoprotein T cell. The figure reveals that most ORIGINAL epitopes lie between residue positions 300 and 365 which is the C-terminal domain (CTD). However, a considerable number of CANDIDATE epitopes are observed between residue positions 50 and 120, which is the N-terminal domain (NTD). A few regions of gaps with fewer epitopes around residue position 200, which has high mutation density, were also observed. From Supplemental Figure S6, it is observed that the most prevalent epitopes were on the C terminal domain.

Immunodominance figure of Nucleoprotein B cell (Supplemental Figure S13) reveals that many CANDIDATE epitopes are found, even in regions where no ORIGINAL epitopes are present. We also observe a gap around residue position 200, which is the linker region. The most prevalent epitopes are again present in the C terminal domain (Supplemental Figure S7).

To better understand the relationship between epitopes and protein functional domains, evaluation of the occurrence of complete sequence identity between epitopes and protein domains was performed. Although epitopes are observed to span the length of Nucleoprotein and Spike glycoprotein, no exact matches were observed between epitopes and full length domain sequence or sub-sequence of any domain sequence on SARS-CoV-2 proteins. All matches contained at minimum a single amino acid mutation or indel. Domains and epitopes were only compared with respect to the same parent protein.

#### 3.4.3. Epitope Localization on Protein Structure

**Figure 4:**
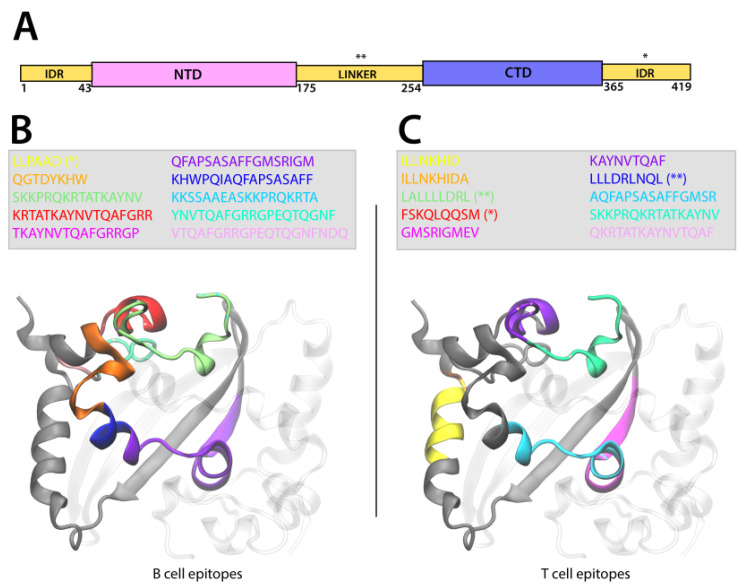
Representation of the localization of the B cell and T cell epitopes on the CTD domain of the Nucleoprotein. (**A**) Scheme of SARS-CoV-2 N domains illustrating the N-term intrinsically disorder region (IDR) followed by the N-terminal domain (NTD), the IDR linker, the C-terminal domain (CTD), and the C-term IDR. (**B**,**C**) The N CTD dimer is represented in New Cartoon format (one monomer is gray and the other is transparent), and the sequence of the B cell (**B**) and T cell (**C**) epitopes is colored according to the legend represented in the figure. The epitope sequence is represented in the legend. The epitopes located in the linker domain are indicated by (**) and those in the C-term IDR by (*). For great clarity, we represented the epitopes in only one monomer.

We analyzed the position of the ten B and T cell epitopes that were most frequently identified in all genomes investigated and observed that 9/10 high frequency B cell and 7/10 high frequency T cell epitopes were localized in the CTD of the N protein. Therefore, in Figure 4B,C only the CTD of the N protein 3D structure is shown, and in Figure 4A, the position of the four epitopes located in the linker or in the C-terminal domain intrinsically disordered region (IDR) of the N protein are indicated. For greater clarity, we represent the position of B and T cell epitopes only on one homodimer and have grayed out the others. The Nucleoprotein CTD structure consists of one $3_{10}$ helix, followed by four α-helices, two β-strands, another α-helix, and another $3_{10}$ helix [13]. In B cells, the ten most frequently identified epitopes are located between the first $3_{10}$ helix and the first β-strand, as shown in Figure 4B. The KKSAAEASKKPRQKRTA (bright blue) and the SKKPRQKRTATKAYNV (green) epitopes are on the first $3_{10}$ helix and overlap. The KRTATKAYNVTQAFGRR (red) epitope is on the first α-helix and is followed by the VTQAFGRRGPEQTQGNFGDQ (pink) epitope. Their sequence includes two other epitopes: the TKAYNVTQAFGRRGP (purple) and YNVTQAFGRRGPEQTQGNF (cyan). The QGTDYKHW (orange) epitope is located between the second and third α-helix, followed by the KHWPQIAQFAPSASAFF (dark blue) and the QFAPSASAFFGMSRIGM (violet) epitopes, which extend half way of the first β-strand. Only one epitope, LLPAAD (yellow), among those represented here is located in the C-terminal IDR (Figure 4A,B). Compared to our observations for the B cell epitopes, the T cells the epitopes are distributed in a discontinuous manner in the C-terminal domain sequence. In addition, two epitopes are located in the linker (LALLLLDRL* (green) and LLLDRLNQL* (dark blue), respectively) and one in the C-term IDR (FSKQLQQSM* (red)) (Figure 4A,C). The SKKPRQKRTATKAYNV (cyan) epitope is on the $3_{10}$ helix and is followed by the KAYNVTQAF* (violet) epitope located on the first α-helix. The sequence of these two epitopes includes that of the QKRTATKAYNVTQAF (pink). The AQFAPSASAFFGMSR epitope (bright blue) is on the third α-helix and partially overlaps the GMSRIGMEV* (purple) epitope, which extends over almost all the first β-strand. The two ILLNKHID* and ILLNKHIDA* epitopes (yellow and orange) located on the last α-helix overlap except for one residue.

**Figure 5:**
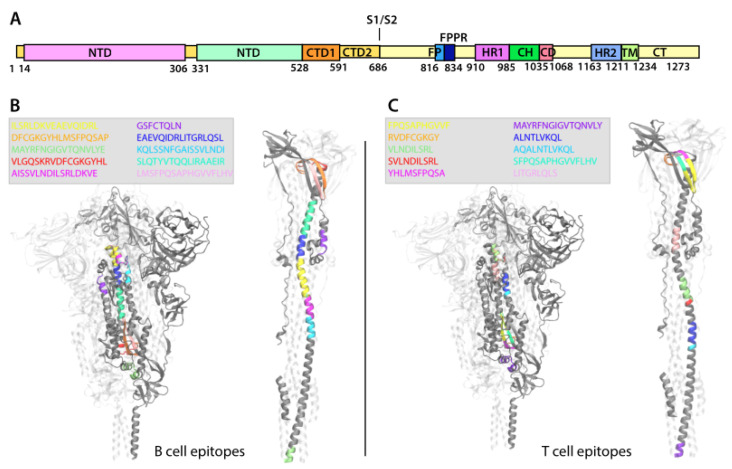
Representation of the localization of the B cell and T cell epitopes on the SARS-CoV-2 Spike glycoprotein in the prefusion and postfusion conformations. (**A**) Scheme of SARS-CoV-2 S1 and S2 units of the S protein and of their domains. (**B**,**C**) The S protein trimer is represented in New Cartoon format (one monomer is gray the other two are transparent) and is shown in the prefusion conformation in the left side of the panels and in the postfusion conformation on the right side of the panels. The sequence of the B cell (**A**) and T cell (**B**) epitopes is shown in the figure legend and is colored accordingly in the S protein structure.

Because the S protein undergoes major conformational changes allowing membrane fusion between the SARS-CoV-2 viral membrane and the host cell, the top ten B cell and T cell epitopes identified after analyzing all genomes have been marked on a 3D representation of the protein. Both prefusion and postfusion states are shown in Figure 5B,C. For clarity, the epitopes are represented only on one homotrimer, with remaining regions grayed out. In addition, Figure 5A shows a schematic representation of the S protein sequence to identify visually the position of the epitopes on the protein.

We observe that the top ten B and T cell epitopes are localized in the S2 subunit of the S protein and, specifically, they are found between residue 902 and 1065, except the B cell epitope GSFCTQLN (violet) located between residue 757 and 764 before the fusion peptide (FP). The B cell epitope MAYRFNGIGVTQNLVYE (green), partially located in the heptad repeat 1 (HR1), is also recognized by T cells, although the two epitope sequences differ for the glutamic acid E, which is absent in the T cell epitope sequence (violet colored sequence in Figure 5C). The five consecutive B cell epitopes KQLSSNFGAISSVLNDI (bright blue), AISSVLNDILSRLDKVE (purple), ILSRLDKVEAEVQIDRL(yellow), EAEVQIDRLITGRLQSL (dark blue), and SLQTYVTQQLIRAAEIR (cyan) are localized between the HR1 domain and almost all the central helix (CH), spanning between residue 964 and 1019 of the S protein. The remaining three B cell epitopes VLGQSKRVDFCGKGYHL (red), DFCGKGYHLMSFPQSAP (orange), and LMSFPQSAPHGVVFLHV (pink) are mostly localized in the connector domain (CD).

As previously observed in the case of the N protein, the T cell epitopes are more widely distributed over the entire length of the S protein S2 subunit. The two epitopes ALNTLVKQL* (dark blue) and VLNDILSRL* (green) are located in the HR1 domain and partially overlap with the other two epitopes AQALNTLVKQL* (bright blue) and SVLNDILSRL* (red). The epitope LITGRLQSL* (pink) is localized entirely in the CH domain. Consecutively located on the CD domain are the following epitopes: RVDFCGKGY* (orange), YHLMSFPQSA* (purple), FPQSAPHGVVF* (yellow), and SFPQSAPHGVVFLHV (cyan).

Additionally, MHC Class I epitopes from the top ten list have been presented on the 3D structure of the S and N proteins in Figure S22 and are denoted with an asterisk (*) in the above section.

## 4. Discussion

During our study, it was observed that 77% of IEDB ground truth data were from SARS-CoV or SARS-CoV-2 (or their parent organisms in taxonomic tree), and 74% of the total epitopes were for Spike glycoprotein or Nucleoprotein (Figure S2). This is a reflection of what was sequenced, and it is important to remember this data bias whilst considering the results described here, as it may influence the observed similarity with respect to other proteins in the SARS-CoV-2 genomes.

In addition, complete sequence homology was found only with respect to identified SARS-CoV-2 and SARS epitopes, whereas no epitopes from other Coronaviridae were found to be present in SARS-CoV-2 genomes when performing an exact sequence match. This result is scientifically interesting because it underscores the similarity between SARS and SARS-CoV-2 genomes and highlights the lack of similarity of SARS and SARS-CoV-2 to other Coronaviridae. This observation has been confirmed in another study of COVID-19 [7]. Our results provide support for the relevance of this work in the face of other emerging viruses, where homology or orthology can be used to rapidly augment our knowledge of epitopes and immunology. The stability of findings across SARS-CoV-2 variants further underscores the extensibility of our method.

Although no exact matches of epitopes were observed with Coronaviridae other than SARS, when considering fuzzy matches or candidate epitopes, we see that fourteen B cell epitopes had parent epitopes found in organisms other than SARS and SARS-CoV-2. These organisms are: Murine hepatitis virus strain JHM, Feline infectious peritonitis virus (strain KU-2), Infectious bronchitis virus, Porcine epidemic diarrhea virus, Murine hepatitis virus strain A59, Avian infectious bronchitis virus (strain M41), Avian infectious bronchitis virus (strain Vic S), and Porcine transmissible gastroenteritis coronavirus strain Purdue. Additionally, 26 candidate T cell epitopes were also found to have parent epitopes belonging to organisms other than SARS and SARS-CoV-2. These organisms are: Feline infectious peritonitis virus (strain KU-2), Human betacoronavirus 2c EMC/2012, Feline infectious peritonitis virus (strain 79-1146), Murine hepatitis virus, and Avian infectious bronchitis virus (strain Vic S). We think that the similarity between regions of SARS-CoV-2 proteins and immune targets of other Coronaviridae might be relevant for investigating viable therapeutics and refining epidemiological models to better estimate effective rate of transmission by accounting for possibly resistant populations. The results shown in Supplemental Figure S1 provide validation for these epitope predictions, which are further confirmed by the fact that the top ten epitopes are found on SARS-CoV-2 Delta variant’s Spike glycoprotein sequence.

We reported in Table 2 and Table 3 a list of the most commonly observed epitopes and marked the T cell MHC Class I epitopes. For brevity, we only discuss the top ten epitopes for each epitope type and protein. Among the most frequent epitopes, we observed that all derived epitopes exhibit parent epitopes belonging to SARS except for one T cell Spike glycoprotein epitope, ISSVLNDILSRLDKVEAEVQ, which had a parent epitope from Feline infectious peritonitis virus (strain 79-114). The bulk of the most commonly seen epitopes were actually original epitopes, i.e., lab confirmed epitopes found as is in SARS or SARS-CoV-2 genomes.

From epitope distributions and quantities in the analyzed genome cohort (Supplemental Figures S16, S18, S19 and S21, respectively), we observed stability of epitope presence across a large set of genomes (62 K). This may provide an advantage from the perspective of multiple downstream analysis, such as therapeutic design, in terms of the host immune response and vaccine development or effectiveness.

In our work, we provide two approaches for selection of epitopes to better understand inter-species homology and relevance for fundamental immunological understanding affecting vaccine design, spread of virus, and development of more accurate epidemiological models:

1. From sequence based clustering (Figure 1), it is possible measure the evolution rate for a cluster by analyzing the ratio of candidate to total epitopes in a cluster. This metric can be useful when evaluating epitope candidates for vaccines and can be used to theoretically predict the probability of change of an epitope solely on sequence homology. Additionally, mapping cluster presence across a genome set adds a dimension for identifying the most suitable epitopes. Additionally, it is possible to filter by host to study changes that might arise from a virus evolving in different hosts. Tracking of major clusters could also enable development of statistical models to estimate a timeline for immune response robustness.
2. By analyzing mutation density regions and immunodominance regions, it is possible to evaluate which segments of proteins may be undergoing the fewest amino acid changes and thus would advise the most stable regions on the protein or those that may be evolving under selective pressure. This type of analysis combined with studying position-based clustering could provide more insight for selection of epitopes, as it would highlight most prevalent epitopes with consideration for their neighbors.

Finally, we analyzed the epitope location on the 3D structure of the N and S proteins. Figure 4 and Figure 5 illustrate the position of the ten most commonly observed epitopes on the 3D representation of the N and the S proteins. In the N protein, 9/10 high frequency B cell and 7/10 high frequency T cell epitopes are localized in the CTD and span different stretches of amino acids, depending on the type of cell. In the S protein, the epitopes are found essentially in the S2 subunit, which overall shares 91% amino acid sequence identity with the SARS-CoV S1 subunit. In addition, the epitopes are primarily found in the HR1, CH, and CD motifs, except for one B cell epitope located in the region upstream of the FP. Compared to prefusion conformation of the S protein, the S postfusion conformation seems to favor a greater exposure of the epitopes to the solvent and thus to antibody binding. The S protein is characterized by a high density glycan surface, which can lead to immune evasion already studied in SARS-CoV-2 and other coronaviruses [59,60,61,62]. X. Fan et al. [63] identified the putative sites of the N-linked glycans shielding the postfusion S protein surface. These sites are completely conserved between SARS-CoV-2 and SARS-CoV. The combined knowledge of the position of the most commonly observed epitopes and the glycan sites is crucial for developing broad-spectrum vaccines and therapeutics, as the S protein is the major determinant for viral transmission. In addition, Supplemental Figure S22 shows presence of MHC Class I epitopes on N and S proteins.

There are important synergies between the work of Grifoni et al. [7] and our study. They performed their work in early 2020 and discussed prediction of immune targets in the absence of lab confirmed targets to speed up vaccine design. Their approach is based on identifying protein and genome similarities to other notable Coronaviridae that have led to previous epidemics and pandemics, including SARS-CoV and MERS. Our work confirms some of their results, such as the similarity between SARS-CoV and SARS-CoV-2. Furthermore, some of the epitopes predicted by Grifoni et al. have also been predicted and observed in our work. Nonetheless, we highlight key differences between the two efforts. Our analysis covers a much larger set of proteins, i.e., 28 K, representing more biological sequence diversity instead of using just reference sequences. In our analysis, we used lab confirmed epitopes for SARS-CoV-2 protein. Finally, during MHC Class I epitope predictions, we observed differences in top HLA binding alleles when using netMHC4.0, but not when MHC classification for the top ten epitopes was investigated manually from IEDB.

## 5. Future Work

1. It is possible to study the proximity of epitopes to more mutation prone regions to eliminate immune targets that otherwise may seem promising.
2. It is possible to also combine the epitope evaluation approaches discussed in Section 4 to design a quantitative metric for evaluating and ranking epitope targets suitable for vaccine development. Selection of stable epitopes is important, as that would enable development of shorter vaccines and may have a positive impact on effectiveness of vaccines because more stable immunotargets may be learned by the host immune system.
3. It would be interesting scientifically to study the presence of the top ten epitopes in vaccine sequences.

## Figures and Tables

**Table 1:** Epitope distribution by protein. * indicates that analysis on Replicase Polyprotein 1a and 1ab is limited Section 2.1.2.

		B Cell Epitopes		T Cell Epitopes	
**Protein Name**	**#Unique Sequences**	**Conserved**	**Candidate**	**Conserved**	**Candidate**
Spike Glycoprotein	4595	50	152	114	29
Nucleoprotein	1737	52	62	65	54
Membrane Protein	285	8	16	18	9
Protein 3a	4	1	1	21	13
Envelope Protein	1	1	0	3	0
ORF6 Protein	109	0	0	5	1
Protein 7a	217	0	1	9	3
Protein 7b	98	0	0	2	1
Protein 9b	7	0	1	0	0
Replicase Polyprotein 1a *	11,509	0	4	3	0
Replicase Polyprotein 1ab *	795	0	0	39	0

**Table 2:** Top 10 most commonly found epitopes in Nucleoprotein. Here, frequency of occurrence is determined by calculating the number of unique genomes an epitope is present in. Homologous epitopes are sister epitopes that cluster together with the epitope in first column when performing clustering by sequence. The cluster change rate indicates the probability of a candidate epitope being found in the cluster. If the cluster consists of only lab confirmed epitopes, the evolution rate will be 0. Start pos is the index of the starting position of epitope on aligned protein sequences, as noted in Section 2.8.1 and in case of every epitope matches the median start position on unaligned sequences. * in front of the sequence indicates that the sequence was found to be a MHC CLass I epitope in majority assays listed on iedb.org.

EPITOPE	NUMBER GENOMES	HOMOLOGOUS EPITOPES and CLUSTER CHANGE RATE	START POS	PARENT EPITOPES
		**Nucleo B cell Epitopes**		
LLPAAD	61,007	0.0	393	N.A
QGTDYKHW	61,004	[] 0.0	293	N.A
SKKPRQKRTATKAYNV	60,996	[SKKPRQKRTATKQYNV] 0.5	254	SKKPRQKRTATKQYNV
KRTATKAYNVTQAFGRR	60,981	[KRTATKQYNVTQAFGRR] 0.5	260	KRTATKQYNVTQAFGRR
TKAYNVTQAFGRRGP	60,980	[TKQYNVTQAFGRRG] 0.5	264	TKQYNVTQAFGRRGP
QFAPSASAFFGMSRIGM	60,978	[] 0.0	305	N.A
KHWPQIAQFAPSASAFF	60,950	[] 0.0	298	N.A
KKSAAEASKKPRQKRTA	60,947	[] 0.0	247	N.A
YNVTQAFGRRGPEQTQGNF	60,941	[VTQAFGRRGPEQTQGNFGDQ] 0.0	267	N.A
VTQAFGRRGPEQTQGNFGDQ	60,920	[YNVTQAFGRRGPEQTQGNF] 0.0	269	N.A
		**Nucleo T cell Epitopes**		
ILLNKHID *	61,092	[ILLNKHIDA] 0.0	350	N.A
ILLNKHIDA *	61,069	[ILLNKHID] 0.0	350	N.A
LALLLLDRL *	61,063	[LLLDRLNQL, GDAALALLLLDRLNQL] 0.0	218	N.A
FSKQLQQSM *	61,063	[] 0.0	402	N.A
GMSRIGMEV *	61,025	[] 0.0	315	N.A
KAYNVTQAF *	61,024	[ TKQYNVTQAF] 0.0	265	N.A
LLLDRLNQL *	61,012	[LALLLLDRL, GDAALALLLLDRLNQL] 0.0	221	N.A
AQFAPSASAFFGMSR	60,996	[ AQFAPSASAFFGMSRIGM] 0.0	304	N.A
SKKPRQKRTATKAYNV	60,996	[SKKPRQKRTATKQYNV] 0.5	254	SKKPRQKRTATKQYNV
QKRTATKAYNVTQAF	60,988	[QKRTATKQYNVTQAF, RQKRTATKAYNVIQAFGRRG] 0.66	259	QKRTATKQYNVTQAF

**Table 3:** Top 10 most commonly found epitopes in Spike glycoprotein sequences. Here, frequency of occurrence is determined by calculating the number of unique genomes an epitope is present in. Homologous epitopes are sister epitopes that cluster together with the epitope in first column when performing clustering by sequence. The cluster change rate indicates the probability of a candidate epitope being found in the cluster. If the cluster consists of only lab confirmed epitopes, the evolution rate will be 0. Start pos is the index of the starting position of epitope on aligned protein sequences, as noted in Section 2.8.1 and in case of every epitope matches the median start position on unaligned sequences. * in front of the sequence indicates that the sequence was found to be a MHC CLass I epitope in majority assays listed on iedb.org.

EPITOPE	NUMBER GENOMES	HOMOLOGOUS EPITOPES and CLUSTER CHANGE RATE	START POS	PARENT EPITOPES
		**Spike B cell Epitopes**		
ILSRLDKVEAEVQIDRL	61,222	[ILSRLDKVEAEVQIDRL] 0.0	979	N.A
DFCGKGYHLMSFPQSAP	61,215	[DFCGKGYHLMSFPQSAP] 1.0	1040	DFCGKGYHLMSFPQAAP
MAYRFNGIGVTQNVLYE	61,213	[MAYRFNGIGVTQNVLYE] 0.0	901	N.A
VLGQSKRVDFCGKGYHL	61,212	[VLGQSKRVDFCGKGYHL] 0.0	1032	N.A
AISSVLNDILSRLDKVE	61,211	[AISSVLNDILSRLDKVE] 0.0	971	N.A
GSFCTQLN	61,209	[GSFCTQLN] 0.0	756	N.A
EAEVQIDRLITGRLQSL	61,205	[EAEVQIDRLITGRLQSL] 0.0	987	N.A
KQLSSNFGAISSVLNDI	61,205	[KQLSSNFGAISSVLNDI] 0.0	963	N.A
SLQTYVTQQLIRAAEIR	61,200	[SLQTYVTQQLIRAAEIR] 0.0	1002	N.A
LMSFPQSAPHGVVFLHV	61,199	[LMSFPQSAPHGVVFLHV] 1.0	1048	LMSFPQAAPHGVVFLHV
		**Spike T cell Epitopes**		
FPQSAPHGVVF *	61,227	[] 0.0	1051	N.A
RVDFCGKGY *	61,225	[] 0.0	1038	N.A
VLNDILSRL *	61,225	[ SVLNDILSRL] 0.0	975	N.A
SVLNDILSRL *	61,224	[VLNDILSRL] 0.0	974	N.A
YHLMSFPQSA *	61,223	[] 0.0	1046	N.A
MAYRFNGIGVTQNVLY	61,216	[] 0.0	901	N.A
ALNTLVKQL *	61,212	[AQALNTLVKQL] 0.0	957	N.A
AQALNTLVKQL *	61,211	[ALNTLVKQL] 0.0	955	N.A
SFPQSAPHGVVFLHV	61,210	[LMSFPQSAPHGVVFLHV] 0.5	1050	N.A
LITGRLQSL *	61,209	[] 0.0	995	N.A

## Data Availability

All epitope data can be obtained directly from iedb.org. All protein sequence data can be obtained from any of the following sources: IBM Functional Genomics Platform (ibm.biz/functional-genomics); Publication regarding FGP’s annotation of SARS-CoV-2 data [46]; Delta S sequences are in Supplemental Data SD1; Reference sequences for N and S are in Supplemental Data SD8. All genome metadata can be obtained from NCBI [47] and GISAID [48] directly. For any additional data or for raw formatted files, please reach out to the corresponding authors.

## Supplementary Materials

The following supporting information can be downloaded at: https://www.mdpi.com/article/10.3390/v14081837/s1, Figure S1: Percent identity of matches between predicted and known epitopes for each cell class. Figure S2: Distribution of epitopes by organism and protein. Figure S3: Clustering of Nucleoprotein T cell epitopes ORIGINAL+CANDIDATE and their occurrence. Figure S4: Clustering of Nucleoprotein B cell epitopes ORIGINAL+CANDIDATE and their occurrence. Figure S5: Clustering of Spike glycoprotein B cell epitopes ORIGINAL+CANDIDATE and their occurrence. Figure S6: Clustermap: position based clustering of Nucleoprotein T cell epitopes ORIGINAL+CANDIDATE. Figure S7: Clustermap: position based clustering of Nucleoprotein B cell epitopes ORIGINAL+CANDIDATE. Figure S8: Clustermap: position based clustering of Spike glycoprotein B cell epitopes ORIGINAL+CANDIDATE. Figure S9: B cell epitope presence on Spike glycoprotein. Figure S10: Mutation density on Spike glycoprotein. Figure S11: Unknown amino Acid (X) presence proportion on Spike glycoprotein. Figure S12: T cell epitope presence on Nucleoprotein. Figure S13: B cell epitope presence on Nucleoprotein. Figure S14: Mutation density on Nucleoprotein. Figure S15: Unknown amino acid (X) presence proportion on Nucleoprotein. Figure S16: Spike glycoprotein epitopes and SARS-CoV-2 genomes. Figure S17: Spike glycoprotein epitopes and protein sequences. Figure S18: Spike glycoprotein epitopes and SARS-CoV-2 genomes. Figure S19: Nucleoprotein epitopes and SARS-CoV-2 genomes. Figure S20: Nucleoprotein epitopes and protein sequences. Figure S21: SARS-CoV-2 genomes vs. Nucleoprotein epitopes. Figure S22: MHC Class I epitopes on Nucleoprotein and Spike glycoprotein T cell.

## References

[1] Sanchez-Trincado J.L., Gomez-Perosanz M., Reche P.A. (2017). Fundamentals and methods for T-and B-cell epitope prediction. J. Immunol. Res..

[2] Wieczorek M., Abualrous E.T., Sticht J., Álvaro Benito M., Stolzenberg S., Noé F., Freund C. (2017). Major histocompatibility complex (MHC) class I and MHC class II proteins: Conformational plasticity in antigen presentation. Front. Immunol..

[3] Prachar M., Justesen S., Steen-Jensen D.B., Thorgrimsen S., Jurgons E., Winther O., Bagger F.O. (2020). Identification and validation of 174 COVID-19 vaccine candidate epitopes reveals low performance of common epitope prediction tools. Sci. Rep..

[4] Lin L., Ting S., Yufei H., Wendong L., Yubo F., Jing Z. (2020). Epitope-based peptide vaccines predicted against novel coronavirus disease caused by SARS-CoV-2. Virus Res..

[5] Bahai A., Asgari E., Mofrad M.R.K., Kloetgen A., McHardy A.C. (2021). EpitopeVec: Linear epitope prediction using deep protein sequence embeddings. Bioinformatics.

[6] Jespersen M.C., Peters B., Nielsen M., Marcatili P. (2017). BepiPred-2.0: Improving sequence-based B-cell epitope prediction using conformational epitopes. Nucleic Acids Res..

[7] Grifoni A., Sidney J., Zhang Y., Scheuermann R.H., Peters B., Sette A. (2020). A sequence homology and bioinformatic approach can predict candidate targets for immune responses to SARS-CoV-2. Cell Host Microbe.

[8] Moreira R.S., Filho V.B., Calomeno N.A., Wagner G., Miletti L.C. (2022). EpiBuilder: A Tool for Assembling, Searching, and Classifying B-Cell Epitopes. Bioinform. Biol. Insights.

[9] Saha S., Raghava G.P.S. (2006). Prediction of continuous B-cell epitopes in an antigen using recurrent neural network. Proteins Struct. Funct. Bioinform..

[10] Yao B., Zhang L., Liang S., Zhang C. (2012). SVMTriP: A Method to Predict Antigenic Epitopes Using Support Vector Machine to Integrate Tri-Peptide Similarity and Propensity. PLoS ONE.

[11] Collatz M., Mock F., Barth E., Hölzer M., Sachse K., Marz M. (2020). EpiDope: A deep neural network for linear B-cell epitope prediction. Bioinformatics.

[12] Andreatta M., Nielsen M. (2016). Gapped sequence alignment using artificial neural networks: Application to the MHC class I system. Bioinformatics.

[13] Peng Y., Du N., Lei Y., Dorje S., Qi J., Luo T., Gao G.F., Song H. (2020). Structures of the SARS-CoV-2 nucleocapsid and their perspectives for drug design. EMBO J..

[14] Lu X., Pan J., Tao J., Guo D. (2011). SARS-CoV nucleocapsid protein antagonizes IFN-b response by targeting initial step of IFN-b induction pathway, and its C-terminal region is critical for the antagonism. Virus Genes.

[15] Duan L., Zheng Q., Zhang H., Niu Y., Lou Y., Wang H. (2020). The SARS-CoV-2 spike glycoprotein biosynthesis, structure, function, and antigenicity: Implications for the design of spike-based vaccine immunogens. Front. Immunol..

[16] Belouzard S., Millet J.K., Licitra B.N., Whittaker G.R. (2012). Mechanisms of coronavirus cell entry mediated by the viral spike protein. Viruses.

[17] Zuniga S., Cruz J.L., Sola I., Mateos-Gomez P.A., Palacio L., Enjuanes L. (2010). Coronavirus nucleocapsid protein facilitates template switching and is required for efficient transcription. J. Virol..

[18] Cong Y., Ulasli M., Schepers H., Mauthe M., V’Kovski P., Kriegenburg F., Thiel V., de Haan C.A.M., Reggiori F. (2020). Nucleocapsid protein recruitment to replication-transcription complexes plays a crucial role in coronaviral life cycle. J. Virol..

[19] Surjit M., Liu B., Jameel S., Chow V.T., Lal S.K. (2004). The SARS coronavirus nucleocapsid protein induces actin reorganization and apoptosis in COS-1 cells in the absence of growth factors. Biochem. J..

[20] Surjit M., Liu B., Chow V.T., Lal S.K. (2006). The nucleocapsid protein of severe acute respiratory syndrome-coronavirus inhibits the activity of cyclin-cyclin-dependent kinase complex and blocks S phase progression in mammalian cells. J. Biol. Chem..

[21] Steuler H., Schröder B., Bürger H., Scholtissek C. (1985). Sequence of the nucleoprotein gene of influenza A/parrot/Ulster/73. Virus Res..

[22] Chang C.K., Hou M.H., Chang C.F., Hsiao C.D., Huang T.H. (2014). The SARS coronavirus nucleocapsid protein–forms and functions. Antivir. Res..

[23] Chen C.Y., Chang C.K., Chang Y.W., Sue S.C., Bai H.I., Riang L., Hsiao C.D., Huang T.H. (2007). Structure of the SARS coronavirus nucleocapsid protein RNA-binding dimerization domain suggests a mechanism for helical packaging of viral RNA. J. Mol. Biol..

[24] Saikatendu K.S., Joseph J.S., Subramanian V., Neuman B.W., Buchmeier M.J., Stevens R.C., Kuhn P. (2007). Ribonucleocapsid formation of severe acute respiratory syndrome coronavirus through molecular action of the N-terminal domain of N protein. J. Virol..

[25] Lin S.Y., Liu C.L., Chang Y.M., Zhao J., Perlman S., Hou M.H. (2014). Structural basis for the identification of the N-terminal domain of coronavirus nucleocapsid protein as an antiviral target. J. Med. Chem..

[26] Szelazek B., Kabala W., Kus K., Zdzalik M., Twarda-Clapa A., Golik P., Burmistrz M., Florek D., Wladyka B., Pyrc K. (2017). Structural characterization of human coronavirus NL63 N protein. J. Virol..

[27] Nguyen T.H.V., Lichiere J., Canard B., Papageorgiou N., Attoumani S., Ferron F., Coutard B. (2019). Structure and oligomerization state of the C-terminal region of the Middle East respiratory syndrome coronavirus nucleoprotein. Acta Crystallogr. D Struct. Biol..

[28] Cubuk J., Alston J.J., Incicco J.J., Singh S., Stuchell-Brereton M.D., Ward M.D., Zimmerman M.I., Vithani N., Griffith D., Wagoner J.A. (2021). The SARS-CoV-2 nucleocapsid protein is dynamic, disordered, and phase separates with RNA. Nat. Commun..

[29] Zinzula L., Basquin J., Bohn S., Beck F., Klumpe S., Pfeifer G., Nagy I., Bracher A., Hartl F.U., Baumeister W. (2021). High-resolution structure and biophysical characterization of the nucleocapsid phosphoprotein dimerization domain from the Covid-19 severe acute respiratory syndrome coronavirus 2. Biochem. Biophys. Res. Commun..

[30] Ye Q., West A.M.V., Silletti S., Corbett K.D. (2020). Architecture and self-assembly of the SARS-CoV-2 nucleocapsid protein. Protein Sci..

[31] Kang S., Yang M., Hong Z., Zhang L., Huanga Z., Chen X., He S., Zhoua Z., Zhoua Z., Chen Q. (2020). Crystal structure of SARS-CoV-2 nucleocapsid protein RNA domain reveals potential unique drug targeting sites. Acta Pharm. Sin. B.

[32] Cai Y., Zhang J., Xiao T., Peng H., Sterling S.M., Walsh R.M., Rawson S., Rits-Volloch S., Chen B. (2020). Distinct conformational states of SARS-CoV-2 spike protein. Science.

[33] Li F. (2016). Structure, Function, and Evolution of Coronavirus Spike Proteins. Annu. Rev. Virol..

[34] Walls A.C., Tortorici M.A., Snijder J., Xiong X., Bosch B.J., Rey F.A., Veesler D. (2017). Tectonic conformational changes of a coronavirus spike glycoprotein promote membrane fusion. Proc. Natl. Acad. Sci. USA.

[35] Duquerroy S., Vigouroux A., Rottier P.J.M., Rey F.A., Bosch B.J. (2005). Central ions and lateral asparagine/glutamine zippers stabilize the post-fusion hairpin conformation of the SARS coronavirus spike glycoprotein. Virology.

[36] Wrapp D., Wang N., Corbett K.S., Goldsmith J.A., Hsieh C.L., Abiona O., Graham B.S., McLellan J.S. (2020). Cryo-EM structure of the 2019-nCoV spike in the prefusion conformation. Science.

[37] Walls A.C., Park Y.J., Tortorici M.A., Wall A., McGuire A.T., Veesler D. (2020). Structure, function, and antigenicity of the SARS-CoV-2 spike glycoprotein. Cell.

[38] Bosch B.J., van der Zee R., de Haan C.A., Rottier P.J. (2003). The coronavirus spike protein is a class I virus fusion protein: Structural and functional characterization of the fusion core complex. J. Virol..

[39] Tortorici M.A., Veesler D. (2019). Structural insights into coronavirus entry. Adv. Virus Res..

[40] Kielian M. (2014). Mechanisms of virus membrane fusion proteins. Annu. Rev. Virol..

[41] Harrison S.C. (2015). Viral membrane fusion. Virology.

[42] Weissenhorn W., Dessen A., Harrison L.J.C.S.C., Skehel J.J., Wiley D.C. (1999). Structural basis for membrane fusion by enveloped viruses. Mol. Memb. Biol..

[43] Lee W.S., Wheatley A.K., Kent S.J., DeKosky B.J. (2020). Antibody-dependent enhancement and SARS-CoV-2 vaccines and therapies. Nat. Microbiol..

[44] Vita R., Mahajan S., Overton J.A., Dhanda S.K., Martini S., Cantrell J.R., Wheeler D.K., Sette A., Peters B. (2019). The immune epitope database (IEDB): 2018 update. Nucleic Acids Res..

[45] Seabolt E., Nayar G., Krishnareddy H., Agarwal A., Beck K.L., Kandogan E., Kuntomi M., Roth M., Terrizzano I., Kaufman J. (2020). IBM Functional Genomics Platform, A Cloud-Based Platform for Studying Microbial Life at Scale. IEEE/ACM Trans. Comput. Biol. Bioinform..

[46] Beck K.L., Seabolt E., Agarwal A., Nayar G., Bianco S., Krishnareddy H., Ngo T.A., Kunitomi M., Mukherjee V., Kaufman J.H. (2021). Semi-Supervised Pipeline for Autonomous Annotation of SARS-CoV-2 Genomes. Viruses.

[47] Benson D.A., Karsch-Mizrachi I., Lipman D.J., Ostell J., Sayers E.W. (2009). GenBank. Nucleic Acids Res..

[48] Elbe S., Buckland-Merrett G. (2017). Data, disease and diplomacy: GISAID’s innovative contribution to global health. Glob. Challenges.

[49] Xu Z., Shi L., Wang Y., Zhang J., Huang L., Zhang C., Liu S., Zhao P., Liu H., Zhu L. (2020). Pathological findings of COVID-19 associated with acute respiratory distress syndrome. Lancet Respir. Med..

[50] Finkel Y., Mizrahi O., Nachshon A., Weingarten-Gabbay S., Morgenstern D., Yahalom-Ronen Y., Tamir H., Achdout H., Stein D., Israeli O. (2021). The coding capacity of SARS-CoV-2. Nature.

[51] Altschul S.F., Gish W., Miller W., Myers E.W., Lipman D.J. (1990). Basic local alignment search tool. J. Mol. Biol..

[52] Karlin S., Altschul S.F. (1990). Methods for assessing the statistical significance of molecular sequence features by using general scoring schemes. Proc. Natl. Acad. Sci. USA.

[53] Comber J.D., Philip R. (2014). MHC class I antigen presentation and implications for developing a new generation of therapeutic vaccines. Ther. Adv. Vaccines.

[54] Apweiler R., Bairoch A., Wu C.H., Barker W.C., Boeckmann B., Ferro S., Gasteiger E., Huang H., Lopez R., Magrane M. (2016). UniProt: The universal protein knowledgebase. Nucleic Acids Res..

[55] Katoh K., Misawa K., Kuma K.I., Miyata T. (2002). MAFFT: A novel method for rapid multiple sequence alignment based on fast Fourier transform. Nucleic Acids Res..

[56] Humphrey W., Dalke A., Schulten K. (1996). VMD – Visual Molecular Dynamics. J. Mol. Graph..

[57] Kouranov A., Xie L., de la Cruz J., Chen L., Westbrook J., Bourne P.E., Berman H.M. (2006). The RCSB PDB information portal for structural genomics. Nucleic Acids Res..

[58] Berman H., Westbrook J., Feng Z., Gilliland G., Bhat T., Weissig H., Shindyalov I., Bourne P. (2020). The Protein Data Bank. Nucleic Acids Res..

[59] Watanabe Y., Allen J.D., Wrapp D., McLellan J.S., Crispin M. (2020). Site-specific glycan analysis of the SARS-CoV-2 spike. Science.

[60] Watanabe Y., Berndsen Z.T., Raghwani J., Seabright G.E., Allen J.D., Pybus O.G., McLellan J.S., Wilson I.A., Bowden T.A., Ward A.B. (2020). Vulnerabilities in coronavirus glycan shields despite extensive glycosylation. Nat. Commun..

[61] Yang T.J., Chang Y.C., Ko T.P., Draczkowski P., Chien Y.C., Chang Y.C., Wu K.P., Khoo K.H., Chang H.W., Hsu S.T.D. (2020). Cryo-EM analysis of a feline coronavirus spike protein reveals a unique structure and camouflaging glycans. Proc. Natl. Acad. Sci. USA.

[62] Walls A.C., Xiong X., Park Y.J., Tortorici M.A., Snijder J., Quispe J., Cameroni E., Gopal R., Dai M., Lanzavecchia A. (2019). Unexpected Receptor Functional Mimicry Elucidates Activation of Coronavirus Fusion. Cell.

[63] Fan X., Cao D., Kong L., Zhang X. (2020). Cryo-EM analysis of the post-fusion structure of the SARS-CoV spike glycoprotein. Nat. Commun..

