# Supplementary figures and images for: Predicting Epitope Candidates for SARS-CoV-2
Source: Viruses. 2022 Aug 21;14(8):1837. doi: 10.3390/v14081837 (PMC9416013; doi:10.3390/v14081837)

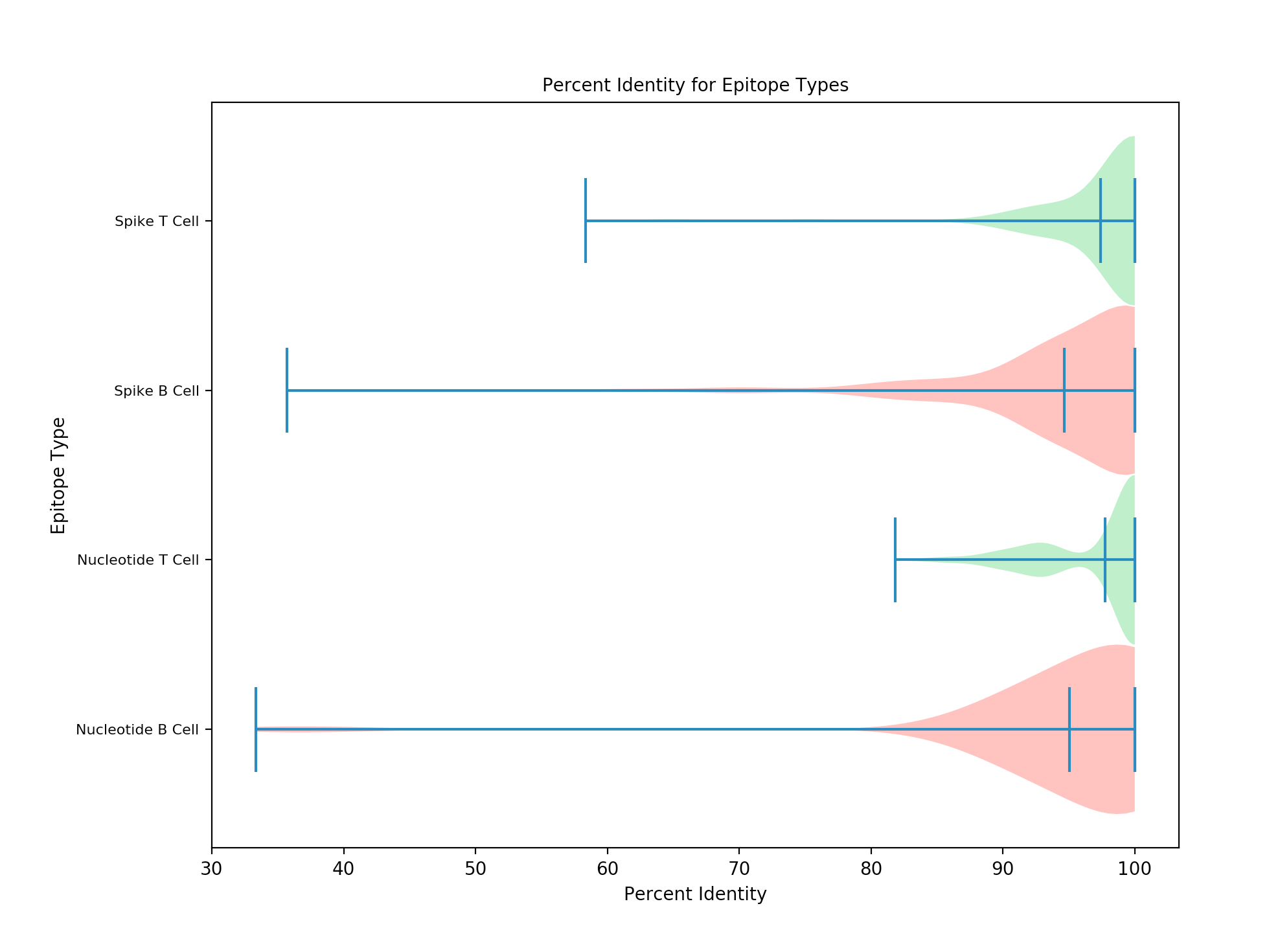

Supplement: Supplementary file 1 [file viruses-14-01837-s001.zip › SupplementalFigures/FigS1.png]

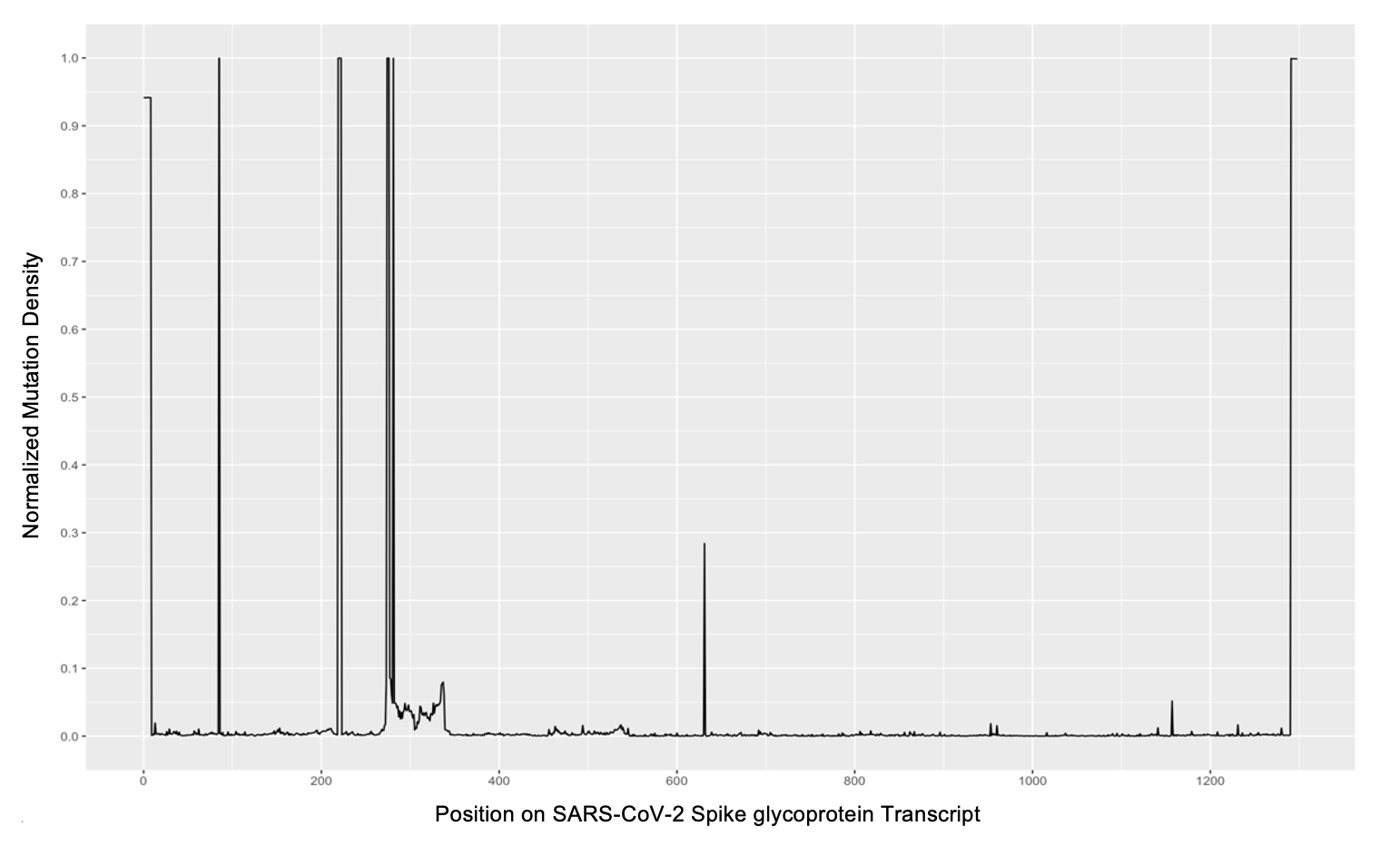

Supplement: Supplementary file 1 [file viruses-14-01837-s001.zip › SupplementalFigures/FigS10.png]

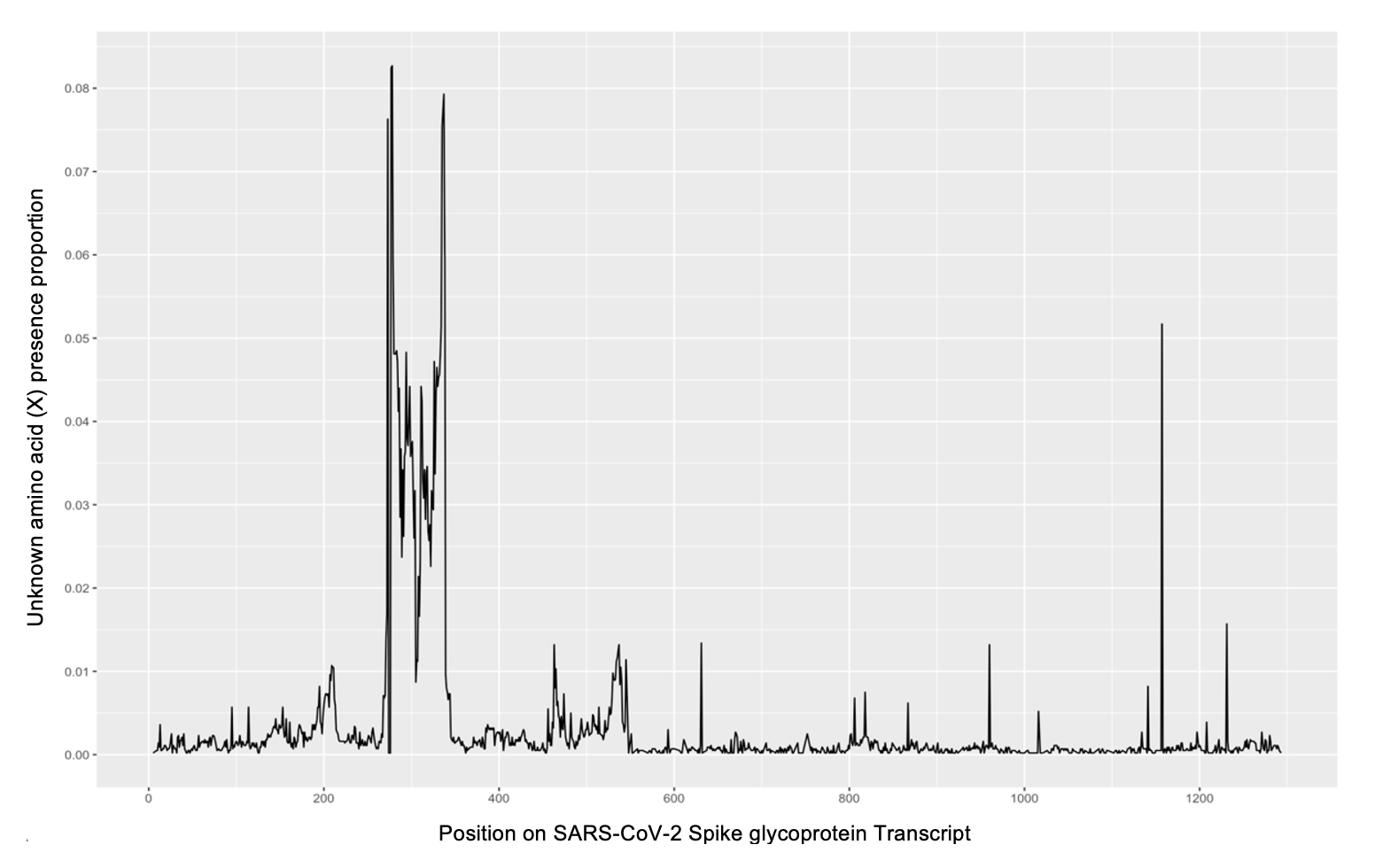

Supplement: Supplementary file 1 [file viruses-14-01837-s001.zip › SupplementalFigures/FigS11.png]

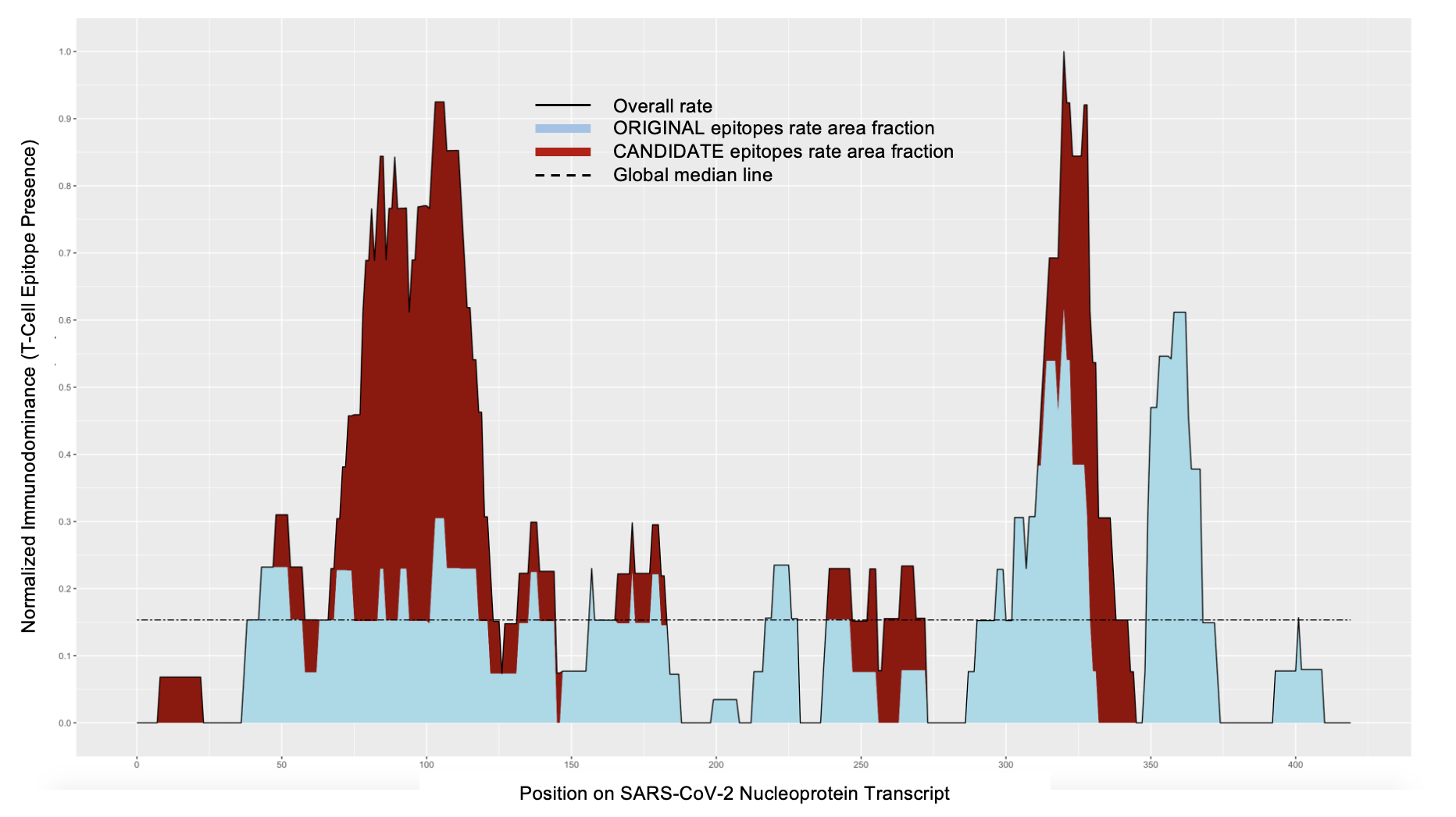

Supplement: Supplementary file 1 [file viruses-14-01837-s001.zip › SupplementalFigures/FigS12.png]

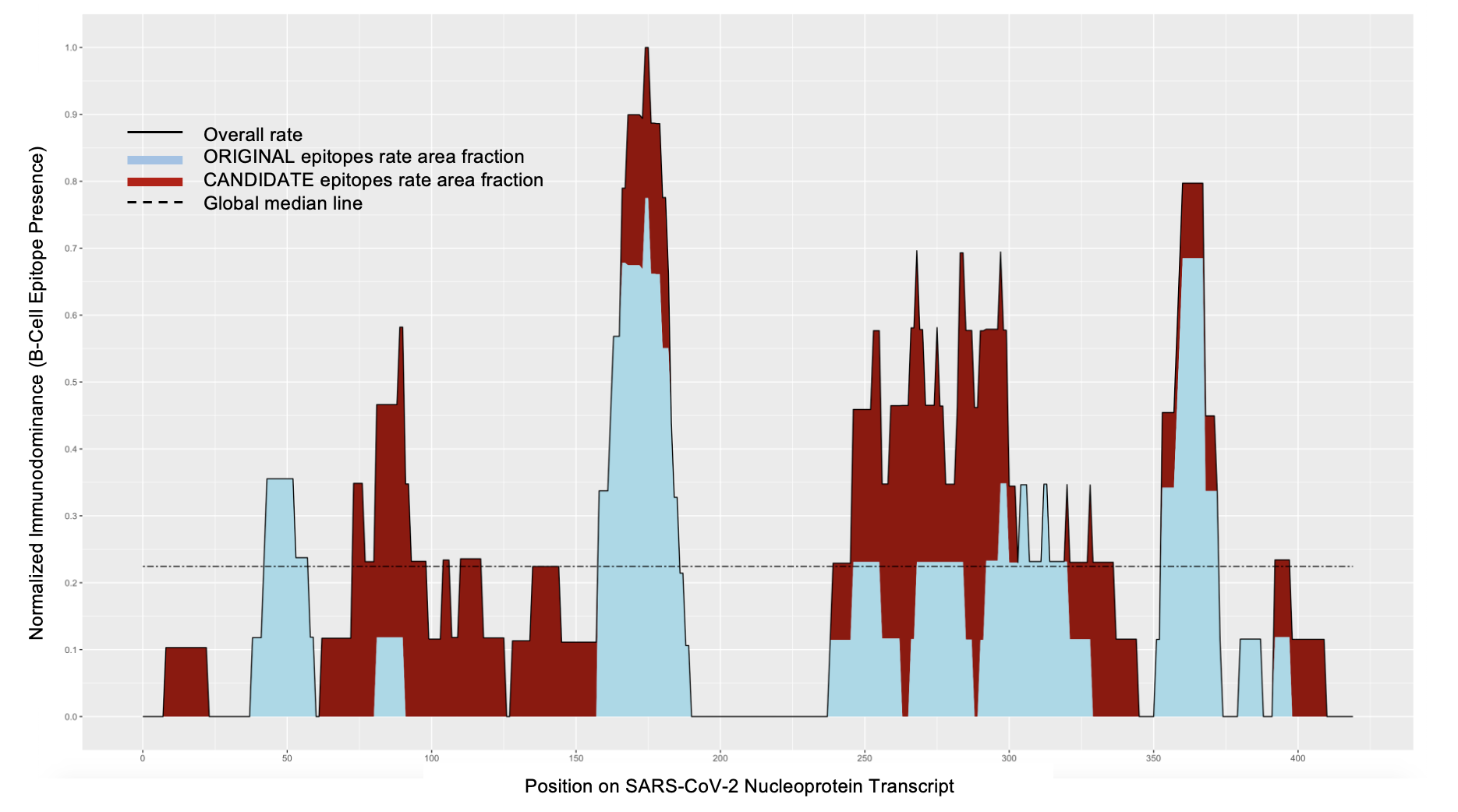

Supplement: Supplementary file 1 [file viruses-14-01837-s001.zip › SupplementalFigures/FigS13.png]

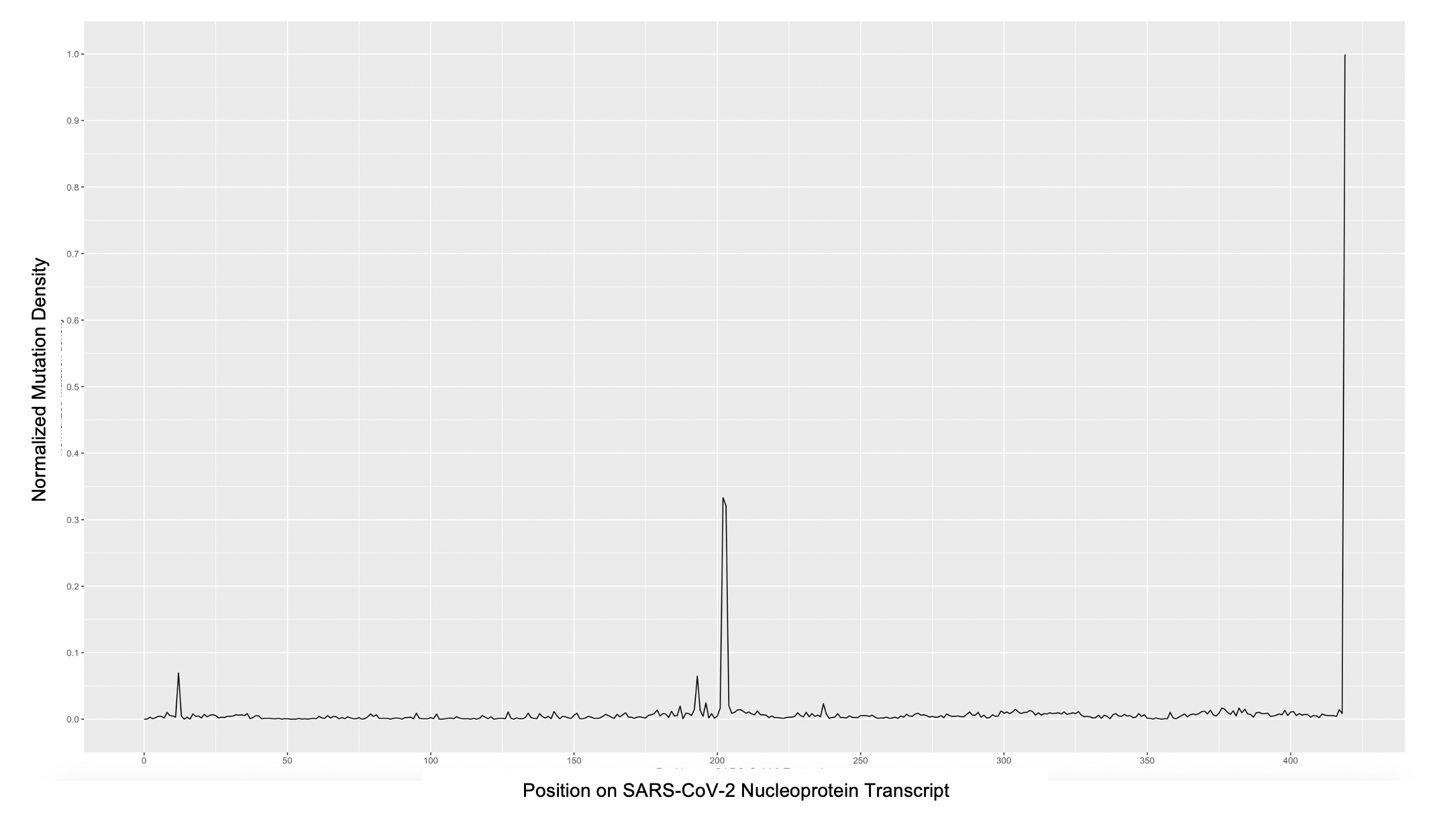

Supplement: Supplementary file 1 [file viruses-14-01837-s001.zip › SupplementalFigures/FigS14.png]

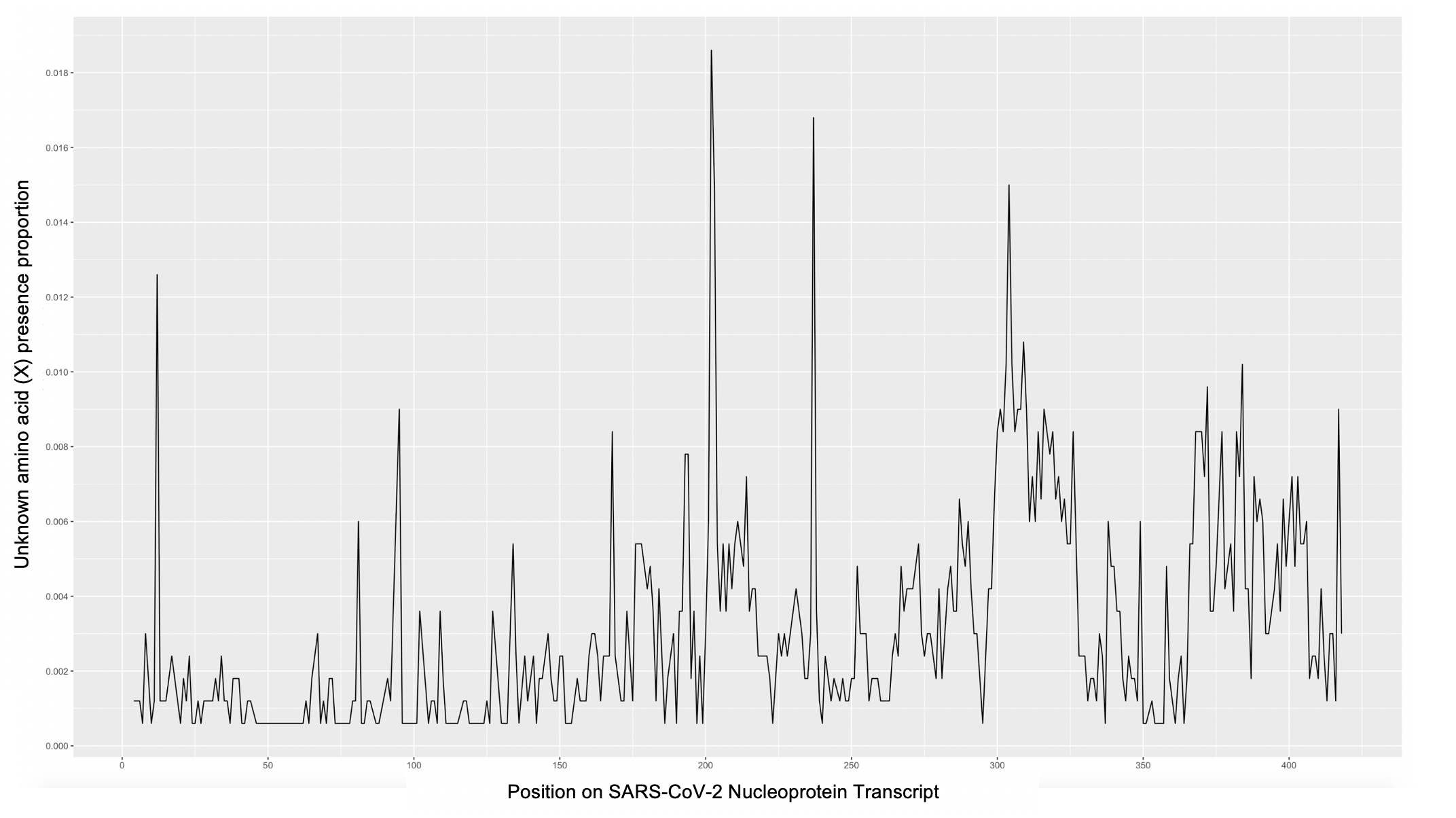

Supplement: Supplementary file 1 [file viruses-14-01837-s001.zip › SupplementalFigures/FigS15.png]

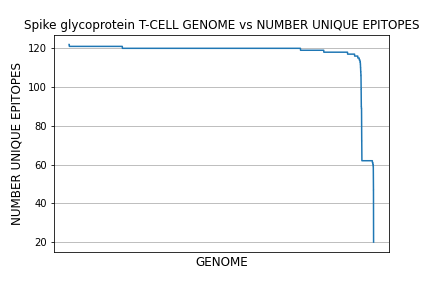

Supplement: Supplementary file 1 [file viruses-14-01837-s001.zip › SupplementalFigures/FigS16.png]

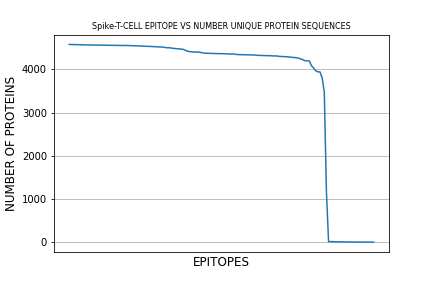

Supplement: Supplementary file 1 [file viruses-14-01837-s001.zip › SupplementalFigures/FigS17.png]

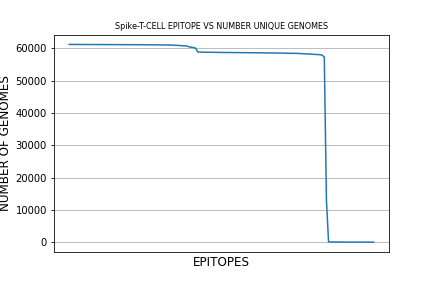

Supplement: Supplementary file 1 [file viruses-14-01837-s001.zip › SupplementalFigures/FigS18.png]

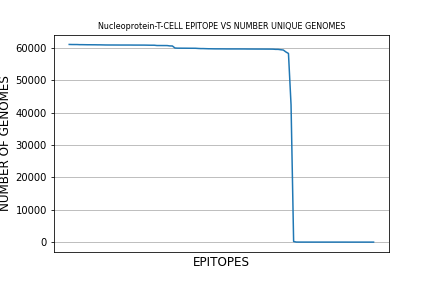

Supplement: Supplementary file 1 [file viruses-14-01837-s001.zip › SupplementalFigures/FigS19.png]

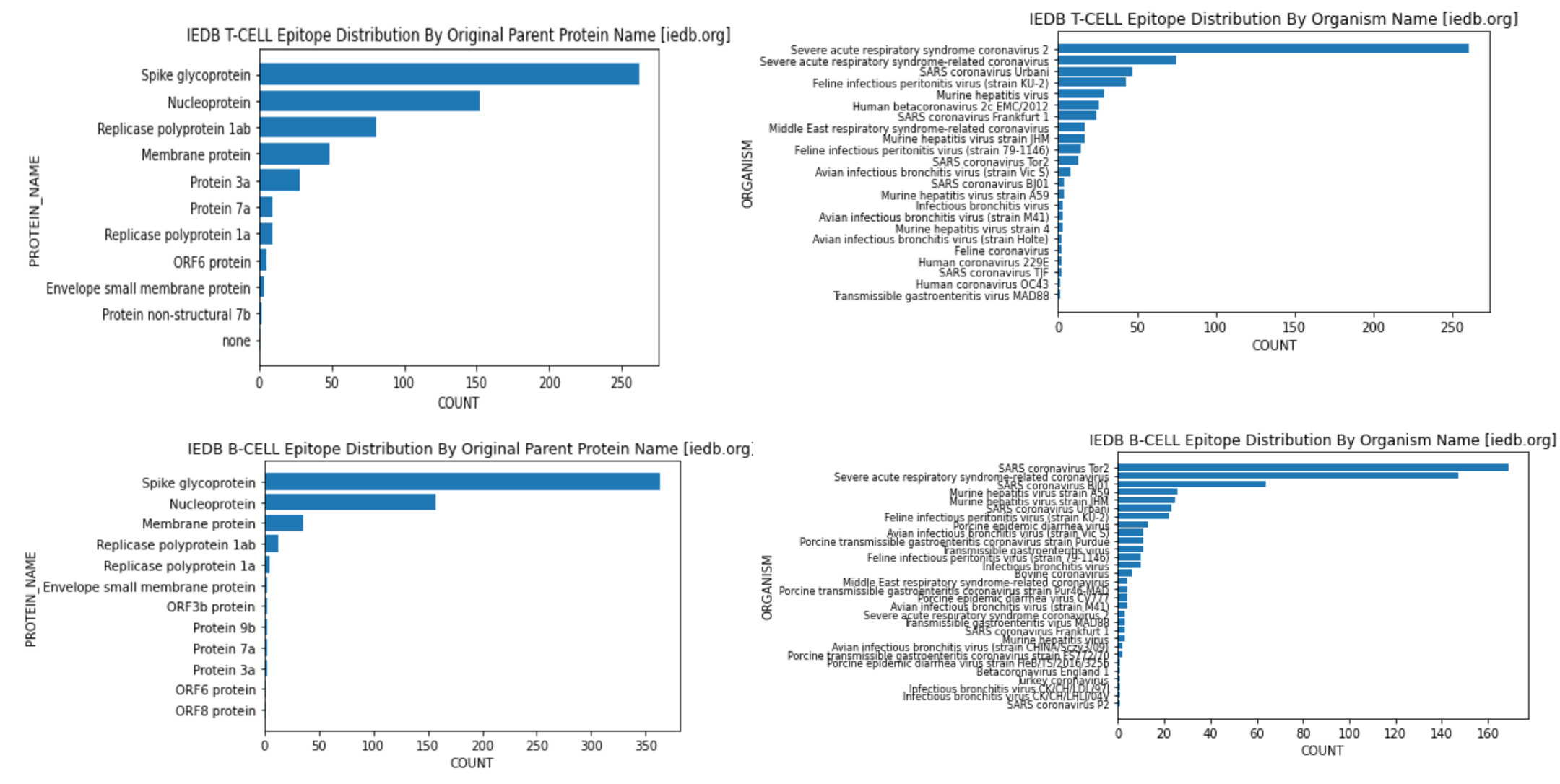

Supplement: Supplementary file 1 [file viruses-14-01837-s001.zip › SupplementalFigures/FigS2.png]

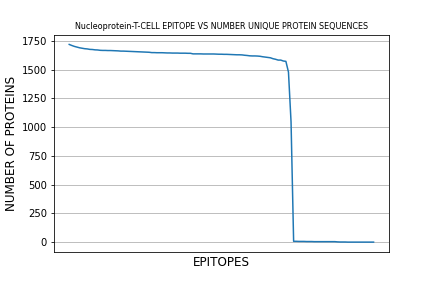

Supplement: Supplementary file 1 [file viruses-14-01837-s001.zip › SupplementalFigures/FigS20.png]

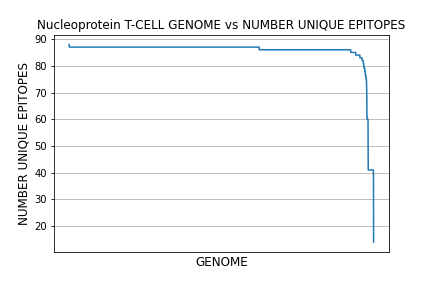

Supplement: Supplementary file 1 [file viruses-14-01837-s001.zip › SupplementalFigures/FigS21.png]

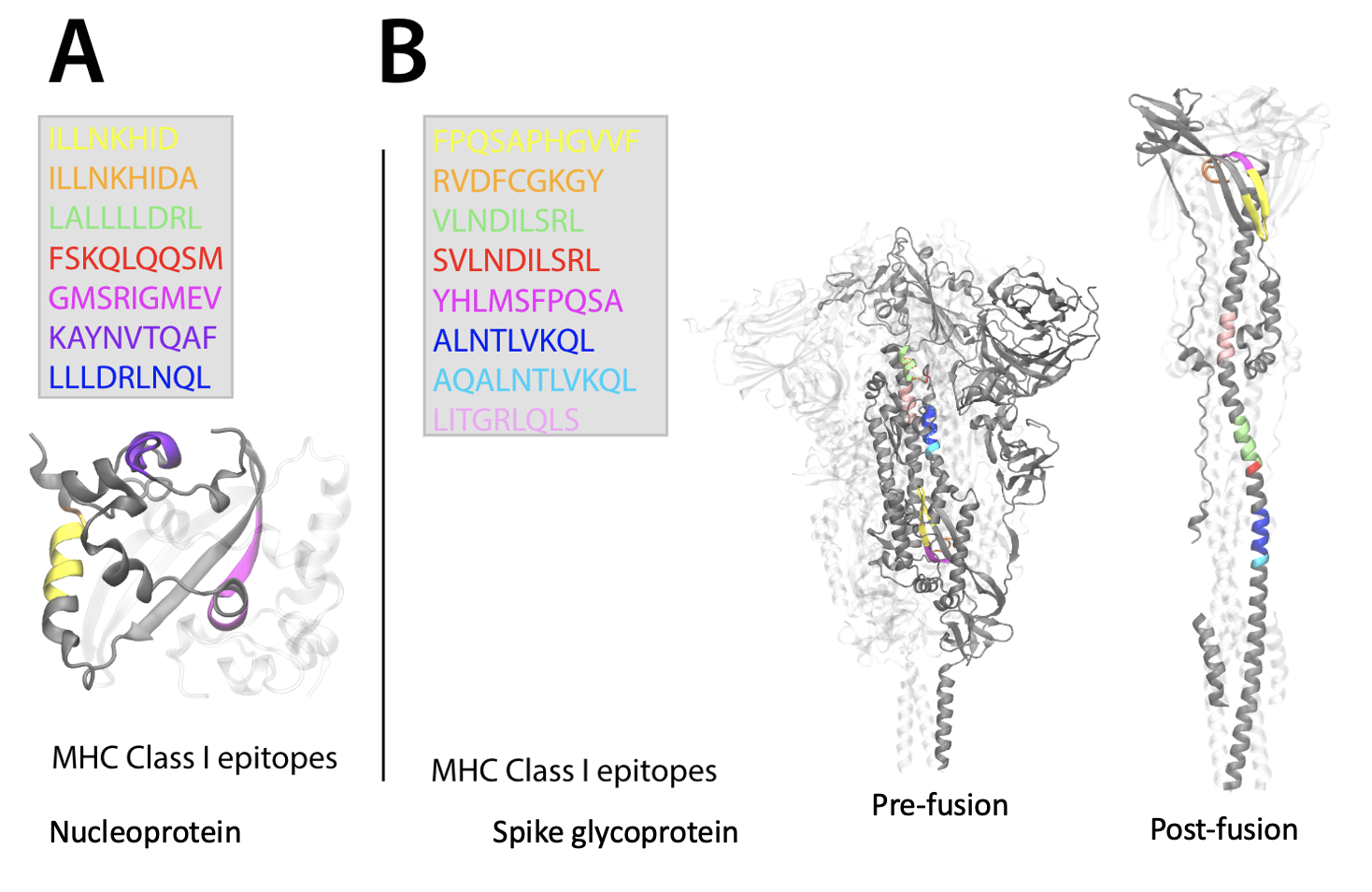

Supplement: Supplementary file 1 [file viruses-14-01837-s001.zip › SupplementalFigures/FigS22.png]

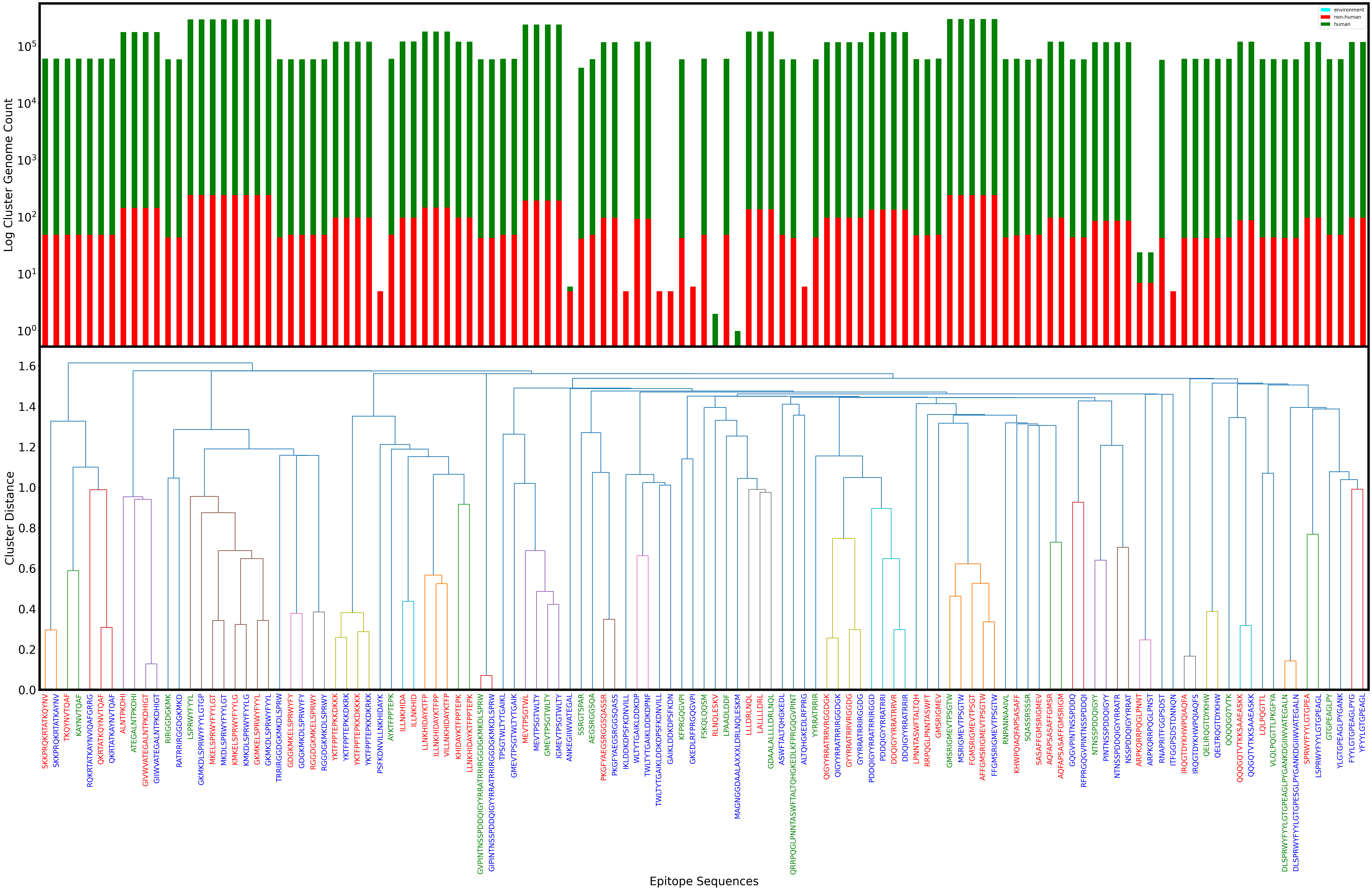

Supplement: Supplementary file 1 [file viruses-14-01837-s001.zip › SupplementalFigures/FigS3.png]

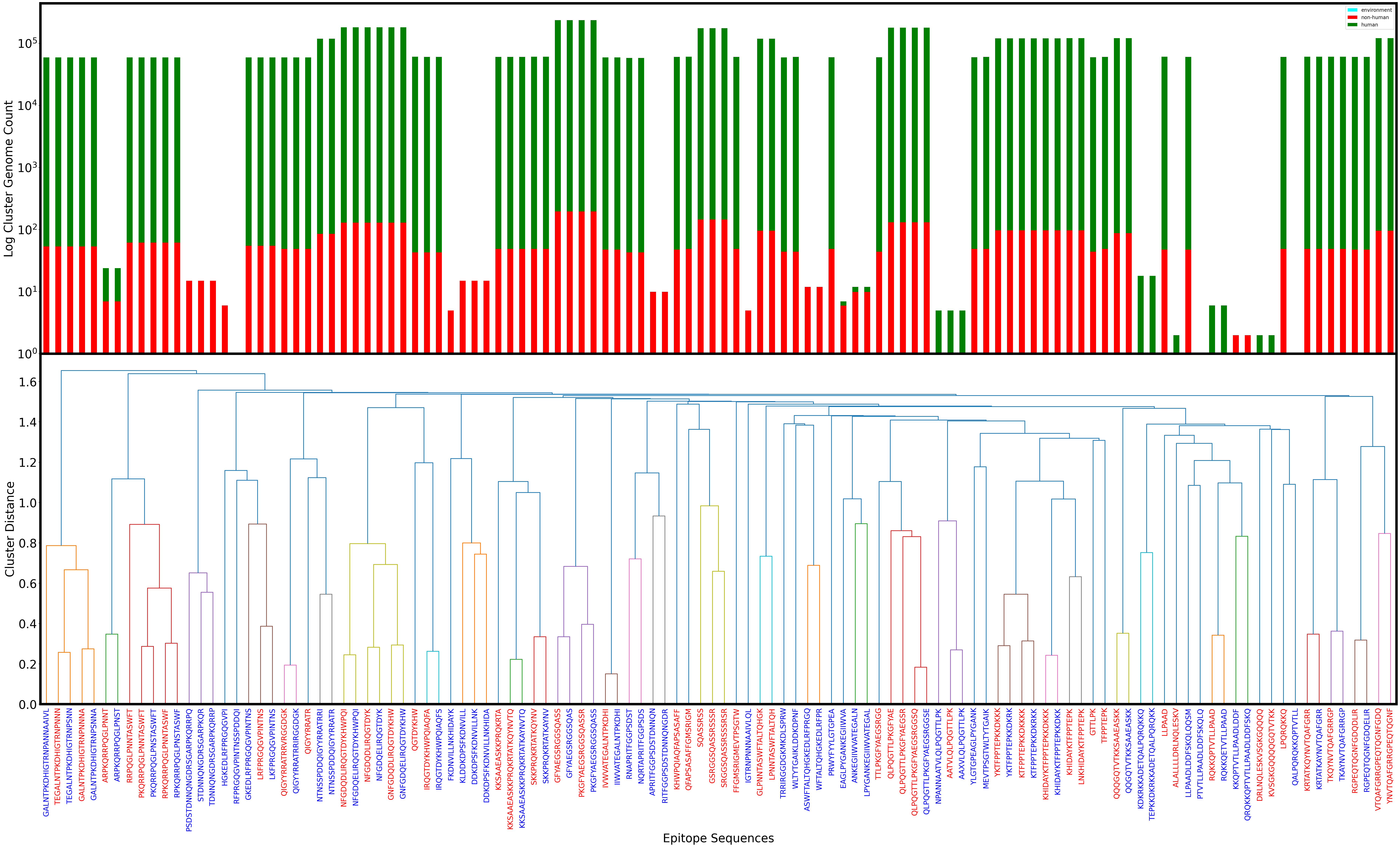

Supplement: Supplementary file 1 [file viruses-14-01837-s001.zip › SupplementalFigures/FigS4.png]

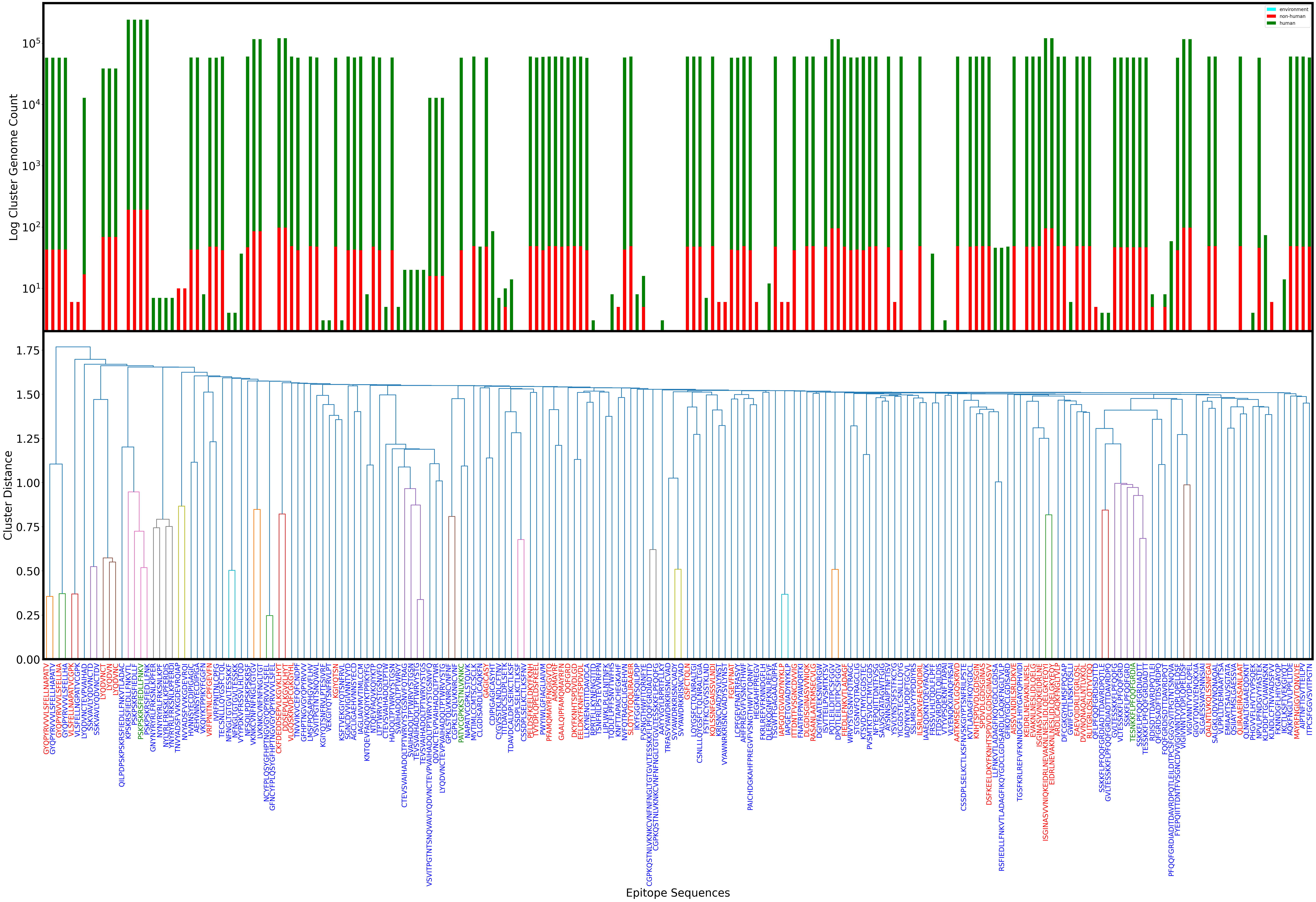

Supplement: Supplementary file 1 [file viruses-14-01837-s001.zip › SupplementalFigures/FigS5.png]

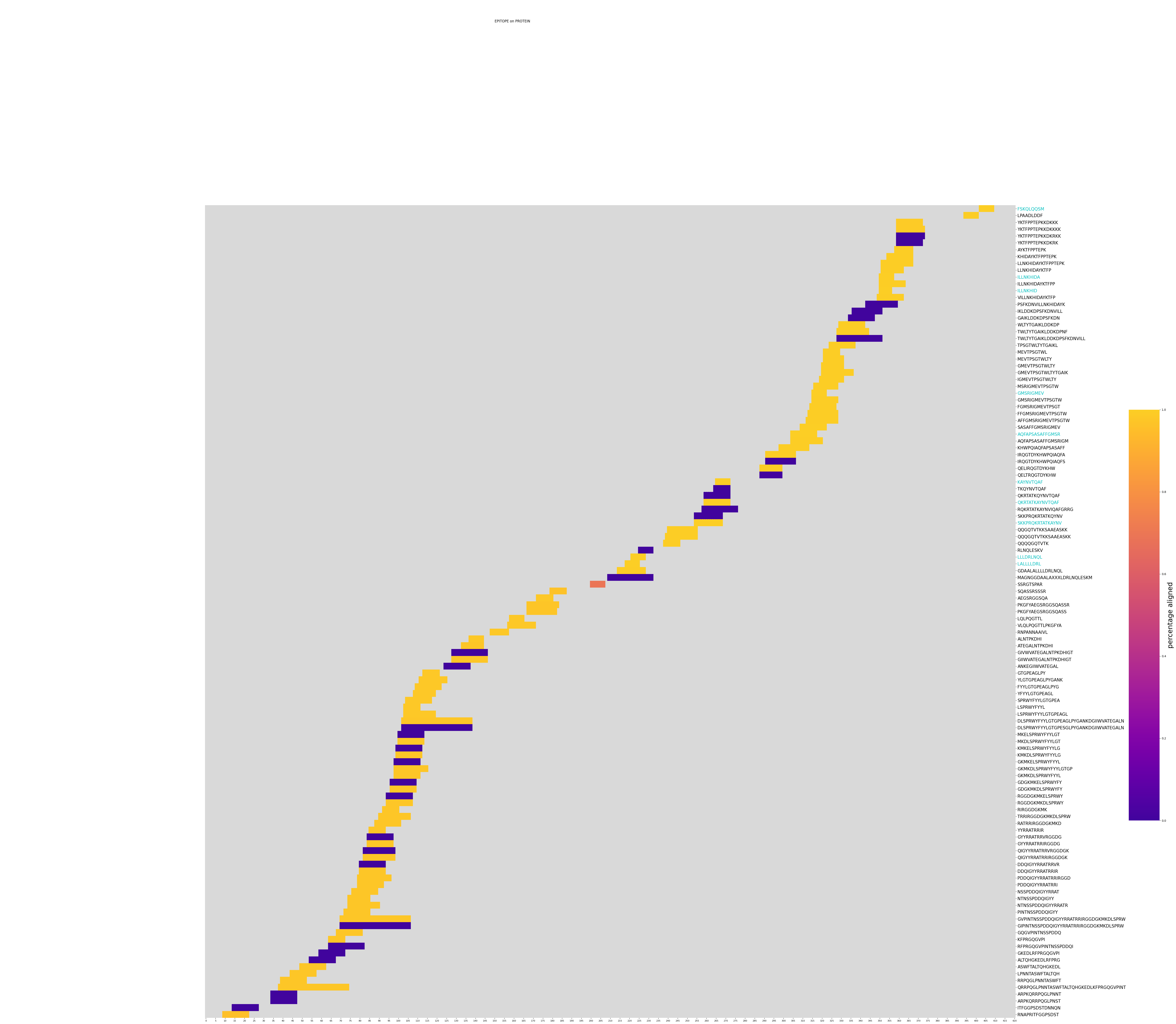

Supplement: Supplementary file 1 [file viruses-14-01837-s001.zip › SupplementalFigures/FigS6.png]

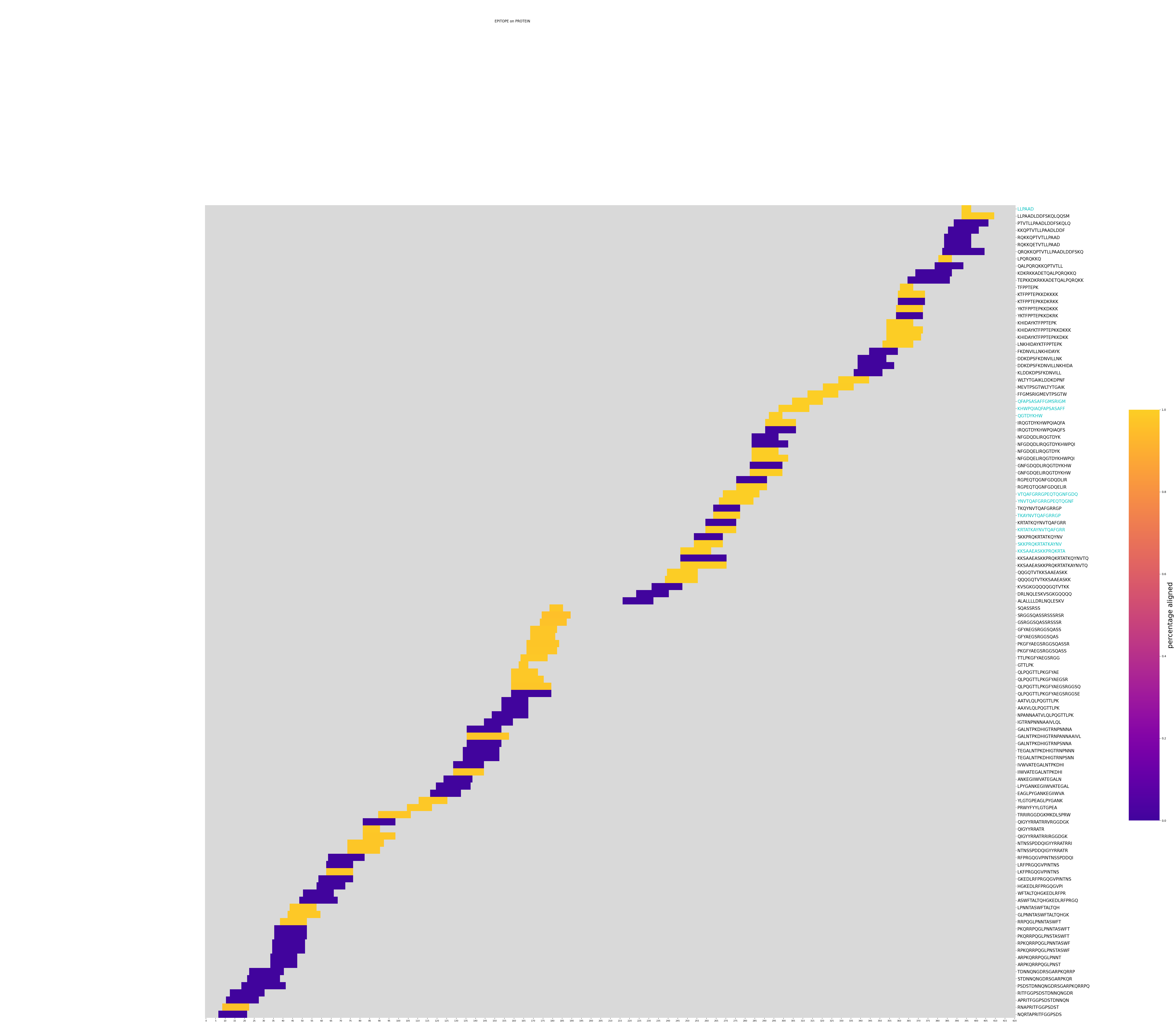

Supplement: Supplementary file 1 [file viruses-14-01837-s001.zip › SupplementalFigures/FigS7.png]

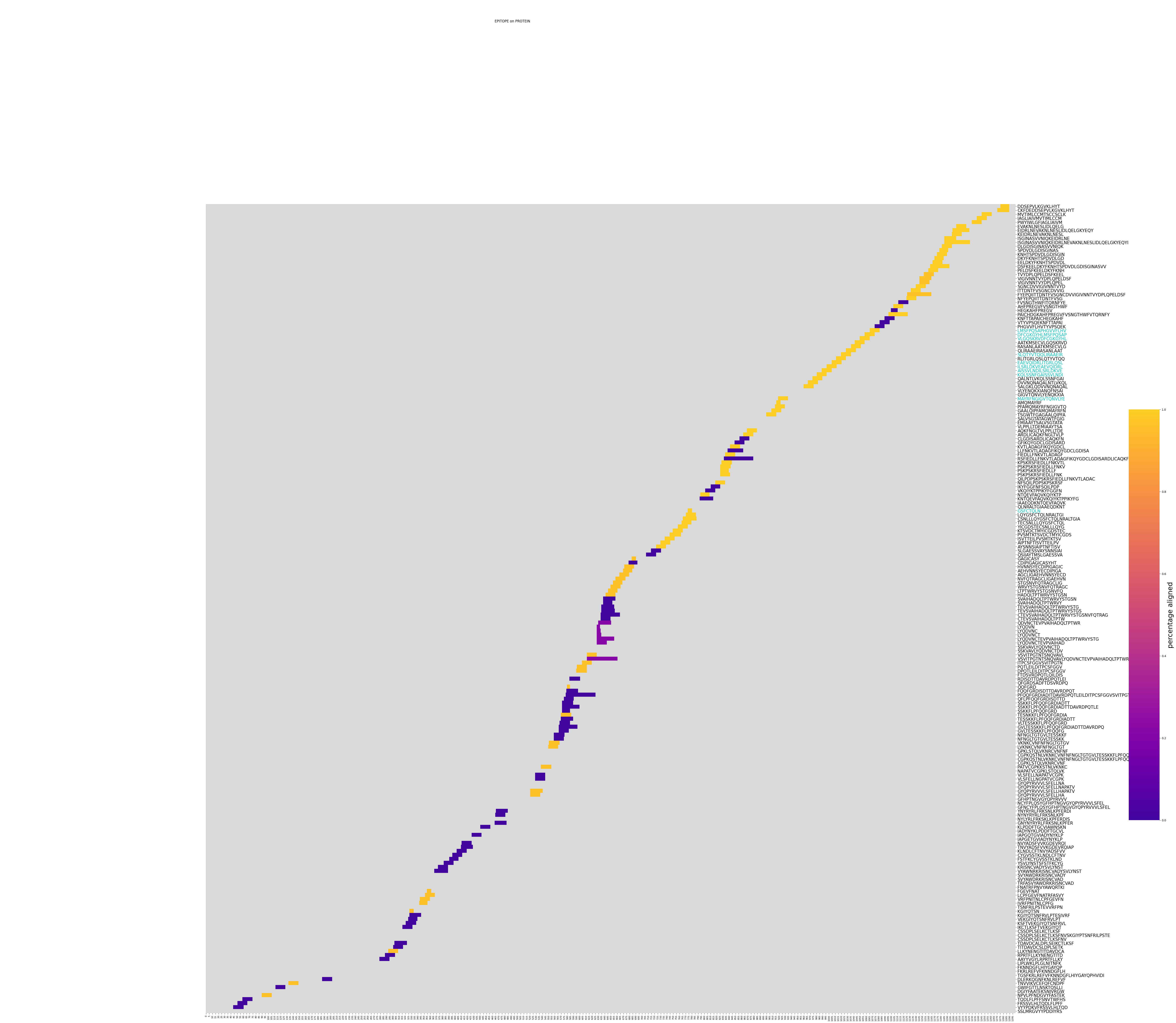

Supplement: Supplementary file 1 [file viruses-14-01837-s001.zip › SupplementalFigures/FigS8.png]

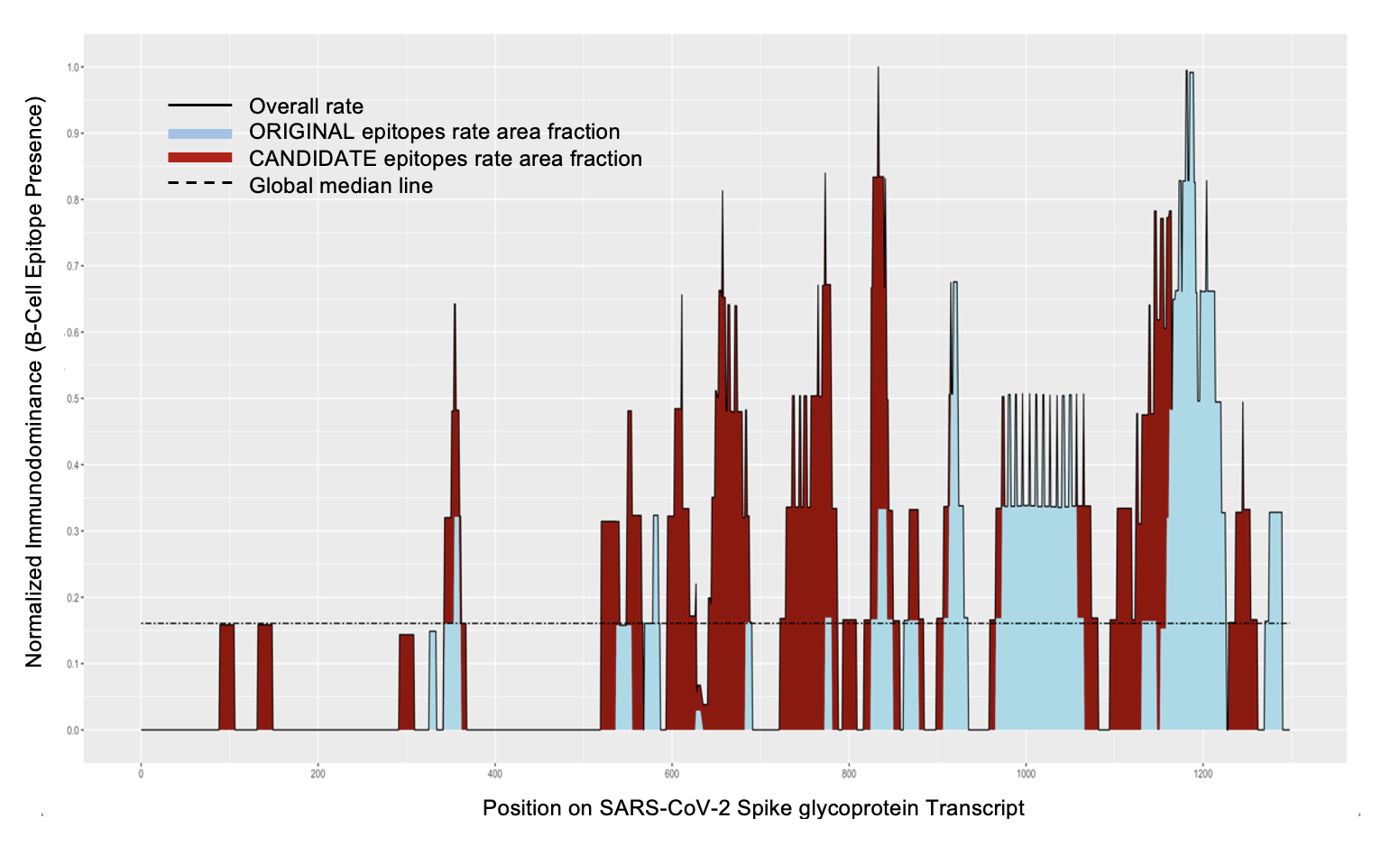

Supplement: Supplementary file 1 [file viruses-14-01837-s001.zip › SupplementalFigures/FigS9.png]
